# Identification of endoplasmic reticulum stress-associated genes and subtypes for predicting risk signature and depicting immune features in inflammatory bowel disease

**DOI:** 10.1016/j.heliyon.2024.e37053

**Published:** 2024-09-01

**Authors:** Ziyu Liu, Zahra Zeinalzadeh, Tao Huang, Yingying Han, Lushan Peng, Dan Wang, Zongjiang Zhou, Diabate Ousmane, Junpu Wang

**Affiliations:** a Department of Pathology, Xiangya Hospital, Central South University, Changsha City, Hunan Province, China; b Department of Pathology, School of Basic Medicine, Central South University, Changsha City, Hunan Province, China; c Ultrapathology (Biomedical electron microscopy) Center, Department of Pathology, Xiangya Hospital, Central South University, Changsha City, Hunan Province, China; d Key Laboratory of Hunan Province in Neurodegenerative Disorders, Xiangya Hospital, Central South University, Changsha City, Hunan Province, China; e National Clinical Research Center for Geriatric Disorders, Xiangya Hospital, Central South University, Changsha City, Hunan Province, China

**Keywords:** Inflammatory bowel disease, Endoplasmic reticulum stress, Predictive model, *TAP1* gene, Immune infiltration, Personalized therapy

## Abstract

Endoplasmic reticulum stress (ERS) becomes a significant factor in inflammatory bowel disease (IBD), like Crohn's disease (CD) and ulcerative colitis (UC). Our research was aimed at identifying molecular markers to enhance our understanding of ERS and inflammation in IBD, recognizing risk factors and high-risk groups at the molecular level, and developing a predictive model on the grounds of based on ERS-associated genes.

This research adopted the least absolute shrinkage and selection operator (LASSO) regression and logistic regression to build a predictive model, and categorized IBD patients into high- and low-risk groups, and then identified four gene clusters. Our key findings included a significant increase in drug target gene expression in high-risk groups, notable discrepancies in immune levels, and functions between high-risk and low-risk groups. Notably, the *TAP1* gene emerged as a strong predictor with the highest diagnostic value (area under the curve [AUC] = 0.941). *TAP1* encodes proteins required for antigenic peptide transfer across the endoplasmic reticulum (ER) membrane, and its potential as a diagnostic marker and therapeutic target is reflected by its overexpression in IBD tissues.

Our study established a new ERS-associated gene model which could forecast the risk, immunological status, and treatment efficacy of patients with IBD. These findings suggest potential targets for personalized therapy and highlight the significance of ERS in the etiology and therapy of IBD. Future studies should explore the therapeutic potential of targeting *TAP1* and other ERS-related genes for IBD management.

## 1 Introduction

Inflammatory bowel disease (IBD) means a group of chronic immune-mediated intestinal inflammatory diseases of unidentified pathogeny, like Crohn's disease (CD) and ulcerative colitis (UC), whose etiology is an intricate pathophysiological combination of genes, intestinal microecology, immune imbalance, and environmental factors [1], which is often manifested as abdominal pain and diarrhea. About 25 % of IBD patients was less than 20 years old when the disease occurred [2]. In Oceania, North America, and various European nations, the morbidity of IBD is more than 0.3 % [3]. During the past recent decades, the morbidity has risen all over the world [4].

The endoplasmic reticulum (ER) can fold polypeptide chain folding and process functional proteins. Some processes contribute to accumulating unfolded or misfolded proteins in the ER, known as endoplasmic reticulum stress (ERS). During the occurrence and progression of IBD, ERS is a significant stress-response component [5]. Triggered by ERS, the pathogeny of IBD is mainly due to the decreased mucosal barrier function in modulating the innate or adaptive immune reactions and intestinal microbiota [6]. Biomarker gene mutations that result in ERS signaling system anomalies highlight the significance of biomarkers in IBD diagnosis [7]. Hence, it is critical to discern molecular markers that boost the ERS function and inflammation in the pathogenesis of IBD, to identify risk factors and high-risk groups at the molecular level early in the disease, and to block disease progression.

ERS can trigger or amplify inflammatory responses in patients with IBD. Therefore, restoration of robust ER stress could be a viable therapeutic target [8]. However, there are currently no biological mechanisms for delivering different types of targeted medications based on ERS levels. We aimed to uncover cellular and molecular pathways to expand a greater comprehension of the IBD phenotype and etiology to personalize and improve therapy as well as to provide predictive indicators designed to guide treatment selection.

## 2 Materials and methods

### 2.1 Acquisition of the IBD dataset and ERS-related genes in the training group

As the training, GSE126124 (platform: GPL6244) set was applied to the prediction model, and the dataset of the mRNA expression profile was supplied by the Gene Expression Omnibus (GEO) database. GSE126124 contains samples from whole blood and colon tissues. For this study, we used colon tissue samples derived from 37 patients with CD, 18 with UC, two with unclassified inflammatory disease, and 21 healthy controls. Corresponding clinical information, including age and sex, was obtained. To create standardized expression values, log2 mean values were generated. The literature contained 785 ERS-related genes [9].

### 2.2 Identification of ERS-related differentially expressed genes (DEGs)

The mRNAs with significant changes in expression between colon tissues from IBD patients and healthy colon tissues in the training group were screened through the “limma” R package. The existing criteria was as below: a regulated *P* value < 0.05 and |log_2_ fold change (FC)| > log_2_1.5. The intersection of 785 ERS-related genes and the DEGs was identified as ERS-related DEGs. The “VennDiagram” R package was adopted to produce a Venn diagram. A heat map was dissected through the R package “pheatmap” to display the expression of ERS-associated DEGs in the IBD and healthy control groups.

### 2.3 Functional enrichment analysis of ERS-related DEGs

Kyoto Encyclopedia of Genes and Genomes (KEGG) pathway enrichment and Gene Ontology (GO) enrichment analyses were implemented through the “clusterProfiler” R package. Gene functions were separated into three types through GO analysis: biological processes (BP), cellular components (CC), and molecular functions (MF). Furthermore, gene set enrichment analysis (GSEA) was employed to construe the ERS-related DEGs enrichment in the KEGG gene sets through the clusterProfiler package. The reference gene set “c2.cp.kegg.v7.4. symbols.gmt” was used.

### 2.4 The least absolute shrinkage and selection operator (LASSO) regression was used to screen the predictive genes of IBD

The fundamental concept behind LASSO regression analysis is reduction in the variable set. The LASSO regression coefficients were constrained using the L1 regularization technique. The most significant variables were chosen for further analysis by compressing the regression coefficients of unnecessary variables to zero and then eliminating them from the model using a penalty function. LASSO regression analysis was implemented through the R package “glmnet” to filtrate genes that could be included in the prediction model among the ERS-related DEGs of IBD, which were referred to as predictor genes.

### 2.5 Construction of ERS-related risk score predictive model for IBD

Logistic regression was used to analyze the predictive genes for IBD, retaining four genes that may be independent indicators, called ERS-related model genes (MGs). A formula for the predictive model is obtained. The risk score per patient was figured out by integrating the regression coefficients of individual genes derived from the logistic regression model and the expression values of each chosen gene. The following formula was used: $\text{Risk}\;\text{score}=\sum_{i=1}^{n}Coefficient\left(mRNAi\right)\times Expression\left(mRNAi\right)$.

### 2.6 Establishment of ERS-related nomogram predictive model for IBD

The nomogram involves the normalization of regression coefficients, which are eventually shown on the number line as risk scores. Using the “rms” R package, we added clinical information (gender and age) to the risk score and generated a 6-variable nomogram.

### 2.7 Evaluation of IBD predictive models

We used the calibration function to resample and create a continuous calibration curve that reflected the discrepancy between the predicted and real outcomes of the nomogram model. The precision and discrimination of the model were rated through area under the curve (AUC) values and receiver operating characteristic (ROC) curves. Decision curve analysis (DCA) was utilized to evaluate the clinical feasibility of the model.

### 2.8 Validation of the risk score predictive model for IBD

GSE75214 (platform: GPL6244) as the validation dataset was utilized to establish the predictive model, including colon tissues from 97 UC patients, eight CD patients, and 11 controls. Moreover, Log2 average values were worked out to gain standardized expression values. The corrected expression data were used to calculate the ERS-related MG expression levels in the validation group samples, and the “pheatmap” R package was adopt to obtain the validation group ERS-associated MG expression heatmap. To figure out the risk scores of the patients, the regression coefficients and expression levels of ERS-related MGs in the validation group samples were merged, and a calibration curve, ROC curve, and DCA graph of the validation group were constructed.

### 2.9 Immune cell infiltration

22 immune cells were infiltrated into each sample. This was determined through the CIBERSORT software. A bar graph was constructed to demonstrate the invasion rate of immune cells from each sample with the immune infiltration filtered through *P* < 0.05. The “ggpubr” R package was utilized to perform immune cells with different invasion percentages in the IBD group by contrast with the control group. The regulatory correlation between ERS-associated MGs and immune cells; besides, the pertinence between immune cells and the patient risk score, was measured through Spearman's rank test. The “ggplot2” R package was adopted to exhibit the results.

### 2.10 Identification of high-risk and low-risk groups and identification of gene clusters

IBD patients were categorized into low- and high-risk groups pursuant to the middle risk score of the GEO cohort composed of training and validation groups. On the grounds of the mRNA expression profiles of four ERS-associated MGs, the “ConsensusClusterPlus” R package was employed to separate samples into gene clusters through consensus clustering. Principal component analysis (PCA) and t-distributed stochastic neighbor embedding (t-SNE) analysis were executed through the “Rtsne” package to assess whether the signature can clearly separate patients into different groups. CIBERSORT was adopted to quantify the levels of 22 immune cell categories infiltration. What's more, a single-sample gene set enrichment analysis (ssGSEA) algorithm was employed to rate differences in immune-related functional enrichment scores across groups.

### 2.11 Single-Cell Portal Analysis

We analyzed CD data titled “PREDICT 2021 paper: CD” and UC data titled “Intra- and inter-cellular rewiring of the human colon” on the Broad Institute online single-cell sequencing database. The CD single-cell data contained 201,883 single-cell transcriptomes from 27 participants, whereas UC single-cell data included 366,650 single-cell transcriptomes from 30 individuals. Then, the t-SNE map of the cell type and four ERS-related MGs were obtained using visualization exploration tools. The expressions of four ERS-associated MGs among various cell types were also demonstrated.

### 2.12 Statistical analysis

Statistical analyses in this research were implemented through the R software (v4.2.3). Relationship analyses were executed through Spearman's correlation coefficients. Contrasts between two groups were carried out through the Wilcoxon rank-sum test; besides, contrasts between three or more groups were conducted through the Kruskal-Wallis test. In addition, *P* value < 0.05 held statistical significance.

## 3 Results

### 3.1 Identification of ERS-related DEGs in IBD

**Fig. 1:**
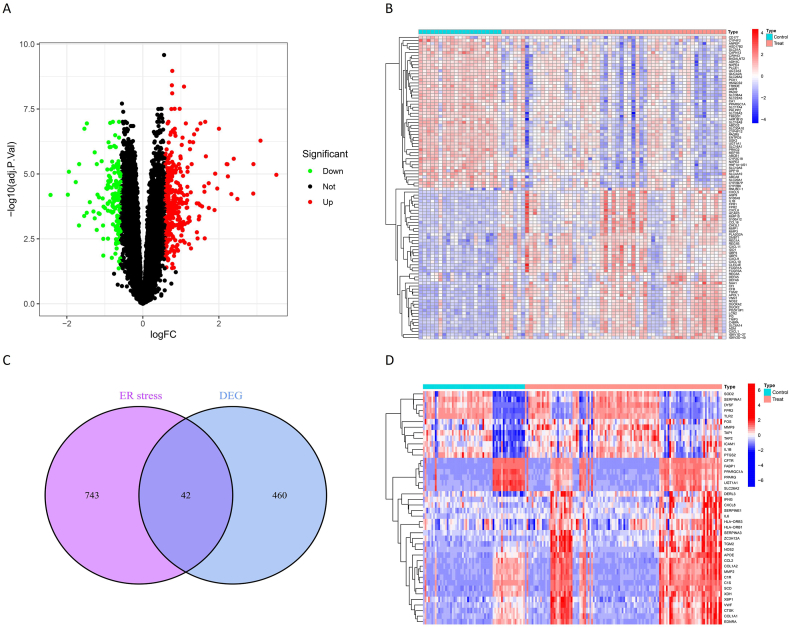
DEG screening. (A) Volcano plot of DEGs. Black, red, and green dots represent genes with no difference, up-regulated genes, and down-regulated genes, respectively. (B) Heatmap of DEGs. The 50 most remarkably up-regulated genes and the 50 most remarkably down-regulated genes are exhibited. Red and blue denote up-regulation and down-regulation, separately. (C) Venn diagram of ERS-related genes vs. DEGs. (D) Heatmap of ERS-associated DEGs. Red and blue denote up-regulation and down-regulation, separately.

We screened 502 DEGs from 23306 genes (Supplementary Table 1). According to the differential gene expression analysis, a volcano plot was used to visualize the DEGs expression (Fig. 1A). Fig. 1B shows the DEGs in the shape of a heat map. The DEGs were combined with 785 ERS-associated genes to attain 42 ERS-associated DEGs (Fig. 1C). Supplementary Table 2 lists the names of the ERS-associated DEGs. The heatmap exhibits the expression of ERS-associated DEGs in patients with IBD and healthy controls (Fig. 1D).

### 3.2 Functional enrichment analysis of ERS-related DEGs

**Fig. 2:**
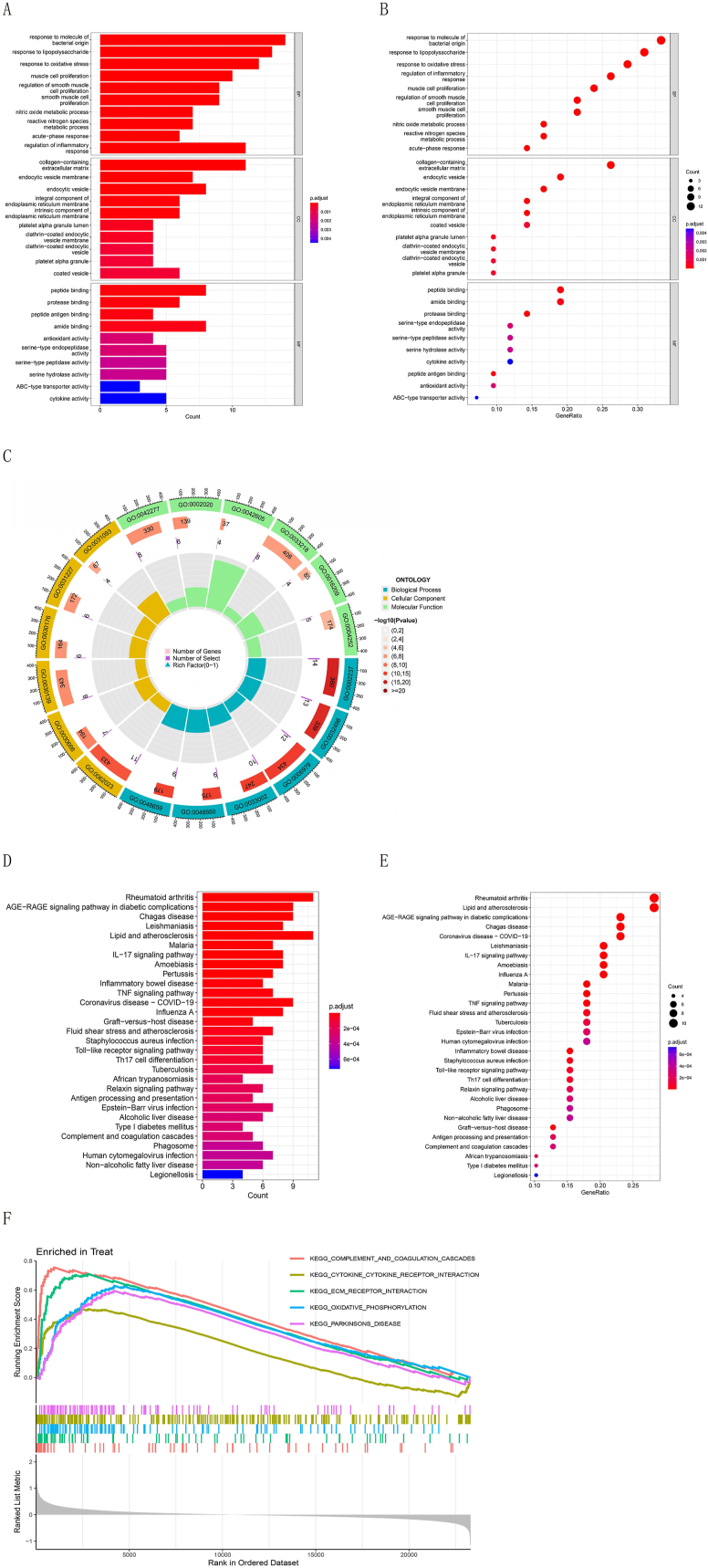
Functional enrichment analysis. (A) Bar plot of GO enrichment analysis. In each class enrichment analysis, the top ten significantly enriched GO terms are displayed. (B) Bubble diagram for GO enrichment analysis. If the gene ratio is higher, the degree of enrichment will be greater. If the q-value is lower, the dot color will be closer to red. If the dots are larger, the more genes will have been identified to be enriched in this pathway. (C) GO circle plot. The top five enriched GO terms from BP, MF, and CC analysis are exhibited. The height of the center column reflected the number of enriched genes. (D) Bar graph of KEGG enrichment analysis. The horizontal axis means the quantity of DEGs in this pathway, and the vertical axis means the descriptive information of the enriched pathways. The top 30 significant enrichment is shown. The colors of the bars correspond to adjusted *P*-values, from small to large, corresponding to blue to red. (E) Bubble diagram for KEGG enrichment analysis. (F) The outcomes of KEGG enrichment analysis were produced through GSEA.

To examine the role of ERS in IBD, a functional annotation of ERS-related DEGs was carried out through GO and KEGG analyses. The genes responded to molecules of bacterial origin, lipopolysaccharides, oxidative stress, and other biological processes (Fig. 2A, B, and C). Cellular components include the collagen-containing extracellular matrix (ECM), endocytic vesicle membrane, integral components of the endoplasmic reticulum membrane, and inherent components of the endoplasmic reticulum membrane. Intersecting genes were rich in many molecular functions, such as peptide binding, protease binding, peptide antigen binding, and antioxidant activity. The key pathways of the DEGs included the *IL-17* signaling pathway, *TNF*, and Toll-like receptor signaling pathways (Fig. 2D and E). Using GSEA, the KEGG terms for complement and coagulation cascades, ECM receptor interactions, and oxidative phosphorylation were identified (Fig. 2F).

### 3.3 Construction of ERS-related risk score predictive model for IBD

**Fig. 3:**
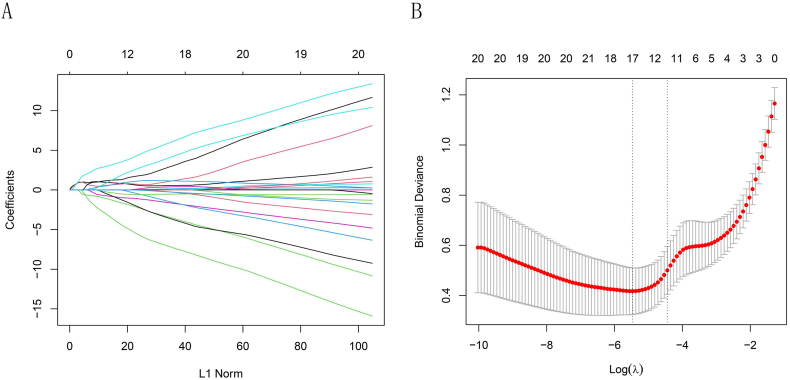
**(**A) Plot of ten-fold cross-validation error rates. (B) LASSO coefficient profiles of 17 predictor genes.

LASSO regression was used to screen 42 ERS-related DEGs to identify predictor genes (Fig. 3A and B). When the number of predictor genes was 17, the mean squared error (MSE) was the smallest, and the prediction of the model constructed by screening the independent variables was the most accurate. The 17 predicted genes are listed in Supplementary Table 3. The Four ERS-related MGs and their corresponding regression coefficients were obtained using logistic regression analysis (Table 1). The predictive model formula was obtained as below: Riskscore= (9.97 × *TAP1* expression level) + (6.35 × *APOE* expression level) + (−15.05 × *CFTR* expression level) + (−5.55 × *MMP9* expression level). In addition, through Statistical Package for the Social Sciences (SPSS) software, no multicollinearity was detected among four genes (Table 2).

**Table 1:** Four ERS-related MGs were obtained through logistic regression. OR, odds ratio; CI, confidence interval.

Gene	Coefficient	OR	OR 95 % CI	*P* value
*TAP1*	9.97	21337.95	56.88–5.29 × 10^9^	0.022
*APOE*	6.35	570.98	13.38–4.29 × 10^5^	0.011
*CFTR*	−15.05	2.90 × 10^−7^	2.34 × 10^−15^–2.62 × 10^−3^	0.019
*MMP9*	−5.55	3.00 × 10^−3^	2.56 × 10^−6^–0.17	0.039

**Table 2:** Collinearity statistics. Collinearity is indicated if the tolerance is < 0.1 or the variance inflation factor (VIF) is > 10.

Gene	Collinearity statistics	
	Tolerance	VIF
*TAP1*	0.452	2.212
*APOE*	0.568	1.761
*CFTR*	0.451	2.219
*MMP9*	0.283	3.535

### 3.4 Establishment of ERS-related nomogram predictive model for IBD

**Fig. 4:**
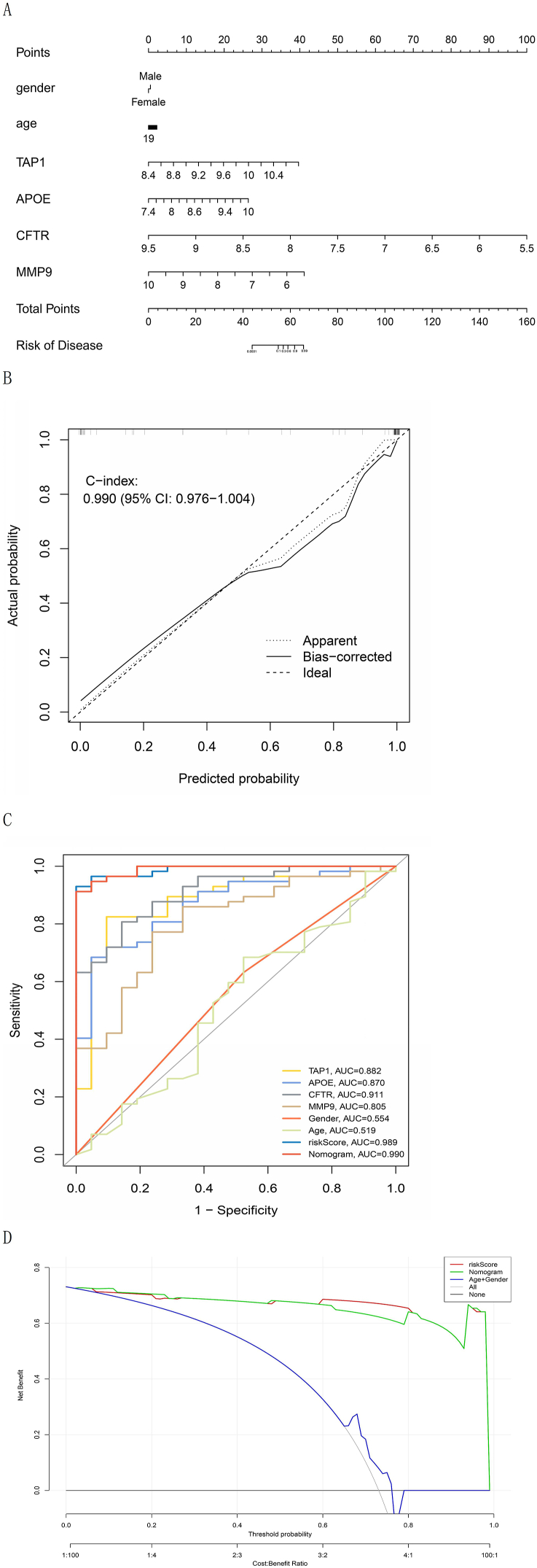
Nomogram constructed using the ERS-related risk score and clinical features. (A) Nomogram of forecasting patients with IBD. (B) Calibration curves of the training group. The deviation between the real and predictive values of the nomogram predictive model was displayed. The concordance index (C-index) was adopted to rate the predictive accuracy of the model by evaluating the consistency between the predictive results and the real observation. (C) The ROC curve of the training group. It is employed to assess the discrimination capacity of the model. The accuracy of the predictive model increases as the area under the ROC curve approaches 1. (D) DCA graph of the training group. To rate the predictive value of the model for clinical applications. The DCA graph shows that both predictive models' threshold probabilities in the training set range 5–95 %, suggesting that both have good clinical applicability.

A nomogram was generated by incorporating sex and age into the ERS-related risk score predictive model (Fig. 4A). High agreement between the predictive and real values was observed in the calibration curves (Fig. 4B). In addition, the clinical efficacy and discrimination capability of the risk score and nomogram predictive models for IBD were assessed (Fig. 4C and D).

### 3.5 Validation of ERS-related predictive models for IBD

**Fig. 5:**
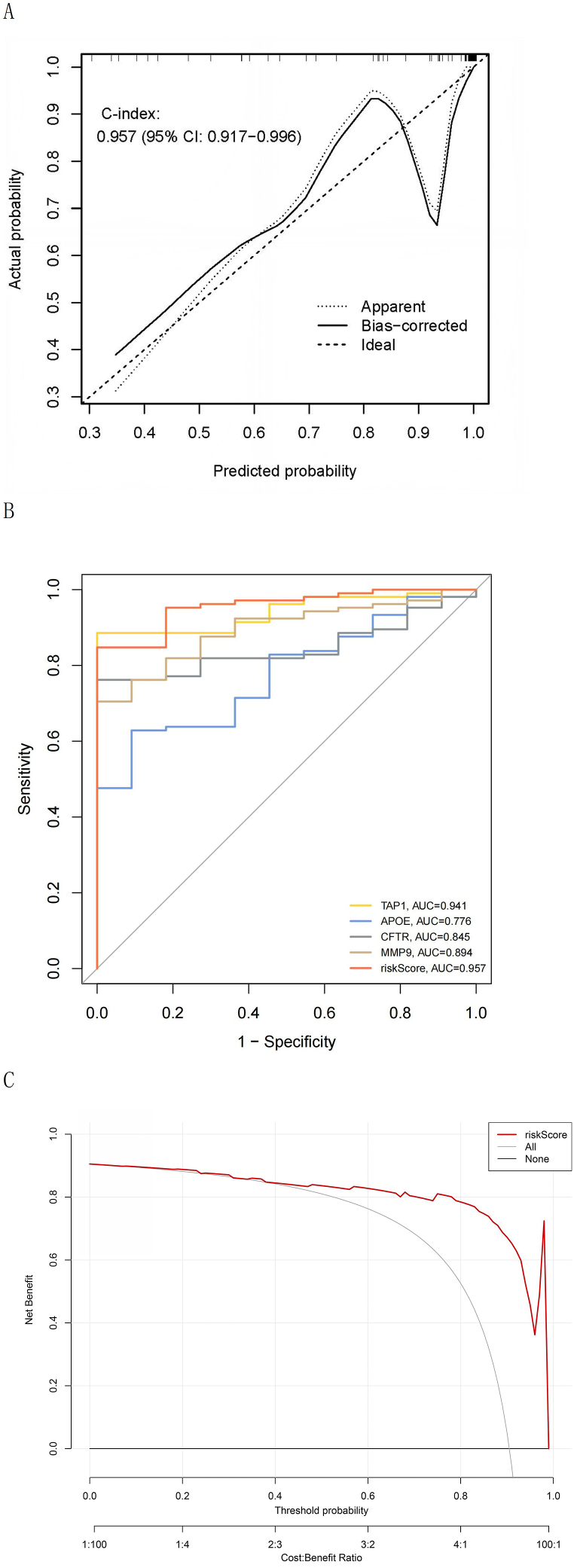
(A) Calibration curve of the validation group. (B) ROC curve. (C) DCA graph. Nomogram showing a greater benefit than the risk score for predicting IBD with a threshold probability ranging 5–95 %.

The calibration curve of the validation group was dissected to rate the accuracy of the model in forecasting the probability of ontogenetic IBD (Fig. 5A). The ROC curve was drawn to rate the its ability to correctly distinguish patients with IBD from the normal population (Fig. 5B), and the DCA graph was drawn to evaluate its clinical practicability (Fig. 5C).

### 3.6 Immune cell infiltration

**Fig. 6:**
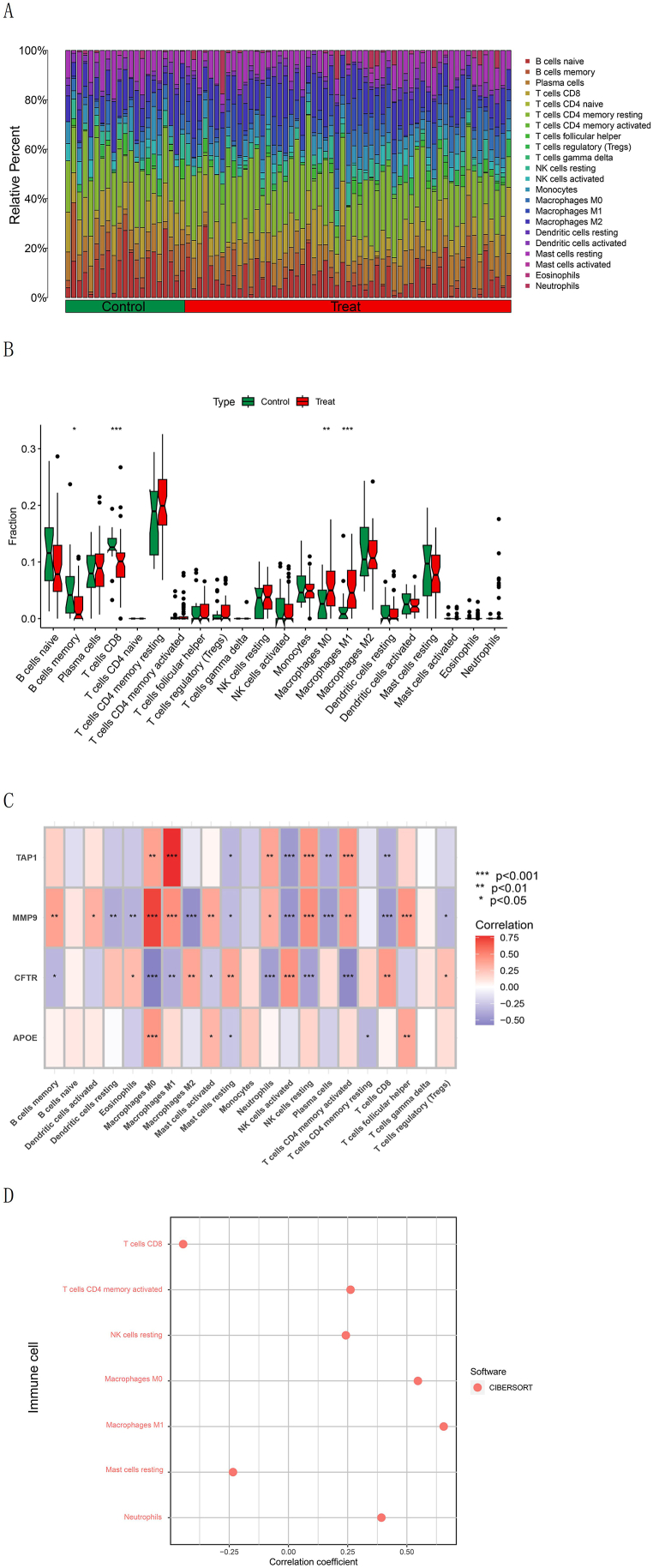
Immune infiltration analysis. (A) Immune infiltration analysis through the CIBERSORT algorithm. (B) Boxplot of 22 types of immune cells in IBD and normal samples. ***P < 0.001, **P < 0.01, and *P < 0.05. (C) Spearman's correlation analysis between ERS-related MGs and immune cells. (D) Spearman's correlation analysis between immune cells and risk score.

Fig. 6A exhibits the rate of 22 categories of immune cells in IBD and normal samples. As shown in Fig. 6B, there existed remarkable differences in the four categories of immune cells between IBD and control samples (*P* < 0.05). Additionally, we discovered a regulatory pertinence between ERS-associated MGs and immune cells (Fig. 6C), and an association between immune cells and risk scores through Spearman's rank correlation test (Fig. 6D).

### 3.7 Risk scores and clustering analysis of patients with IBD

**Fig. 7:**
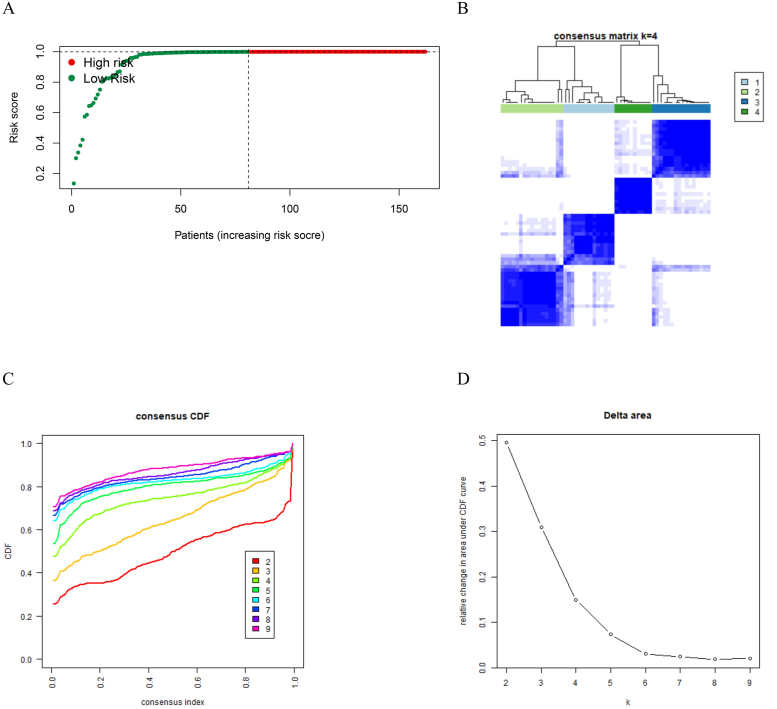
Risk scores and clustering. (A) The distribution and median value of the risk scores in the GEO cohort. The data points are color-coded, with red dots denoting high-risk patients and green ones denoting low-risk patients. The vertical dashed line stands for the median risk score, effectively separating the high-risk group from the low-risk group. Patients on the left of the line, with lower-risk scores, are considered as low-risk, whereas those on the right, with higher risk scores, are classified as high-risk. (B) The consistency matrix heat map. The consensus matrix heat map was generated with k = 4, indicating the optimum number of clusters for categorizing the IBD samples. Each cell in the matrix represents the consensus probability between pairs of samples, with values ranging 0–1, where a value approaching 1 means high consensus (samples are consistently clustered together), and a value approaching 0 denotes low consensus (samples are rarely clustered together). (C) Variation in the area under the CDF plot (k = 2–9). Each curve corresponds to a different number of cluster k, as depicted by the legend on the right of the plot. A flatter curve stands for a more stable clustering solution. (D) Delta area plot for determining optimal number of clusters. The X-axis means the quantity of clusters k, and the Y-axis means the comparative change in the area under the CDF curve. Each point on the plot corresponds to a specific k value, and the line connects these points to illustrate the trend in the comparative change of the area under the CDF curve as k increases. The plot reveals a sharp decrease in the comparative change in area from k = 2 to k = 4, after which the curve flattens out, indicating smaller changes in the area for k > 4. This pattern suggests that k = 4 is the optimum number of clusters, as increasing the clusters beyond this point does not significantly enhance the stability of the clustering solution.

IBD patients were classified into low-risk (n = 81) and high-risk (n = 81) groups in light of the median risk score (risk score = 0.9991625225) in the GEO cohort composed of the training and validation groups (Fig. 7A). This clear separation highlights the efficacy of the risk score model in distinguishing between high- and low-risk patients, demonstrating a remarkable difference between the two groups in view of the median risk score. Fig. 7B shows that four ERS-related MGs effectively classified the IBD samples into four distinct clusters, demonstrating a clear separation between the different groups. This classification allowed for a more detailed understanding of the heterogeneity within the population of IBD patients. Fig. 7C shows that the curve for k = 4 has a relatively flatter distribution compared to the other k values, suggesting that k = 4 could be the optimum number of clusters for this dataset. Fig. 7D shows the comparative change in the area under the cumulative distribution function (CDF) curve for discrepant values of k (ranging 2–9) in the consensus clustering analysis of patients with IBD.

### 3.8 Dimensionality reduction analysis of patients with IBD

**Fig. 8:**
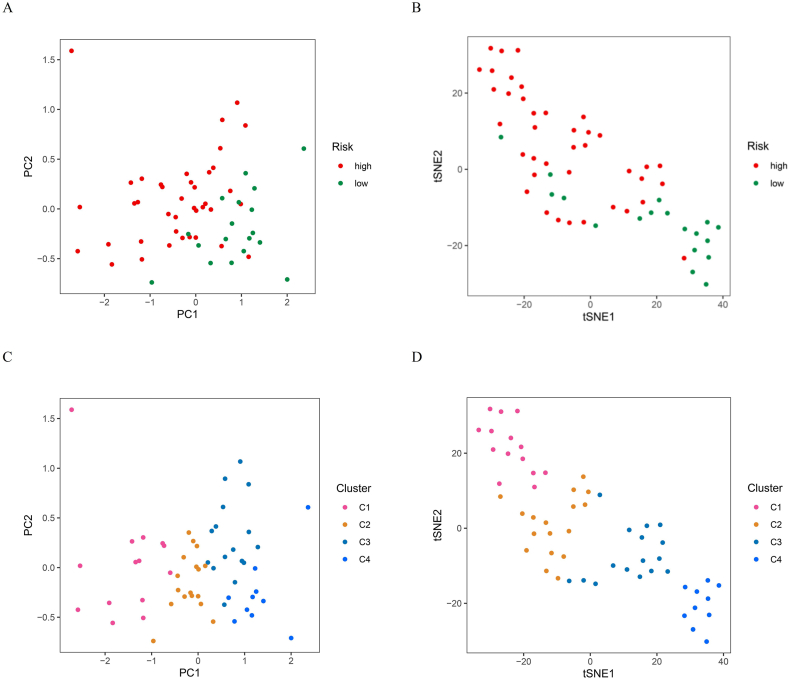
Dimensionality reduction analysis. (A) PCA analysis under high-risk and low-risk groups. The plot shows that the PCA can effectively discriminate between high-risk (red dots) and low-risk patients (green dots). (B) t-SNE analysis under high-risk and low-risk groups. The t-SNE plot reveals a distinct separation between high-risk and low-risk patients. (C) PCA analysis under four gene clusters. PCA plot showing distinct groupings for each cluster, with Cluster 1 (pink), Cluster 2 (orange), Cluster 3 (blue), and Cluster 4 (cyan). (D) t-SNE analysis under four gene clusters. The t-SNE plot confirms the separation of the clusters identified through the PCA analysis, with each cluster forming a distinct group.

PCA and t-SNE were carried out to assess the discrimination between high-risk and low-risk patients as well as between different gene clusters. Fig. 8A shows the PCA results of the high- and low-risk groups. The plot demonstrates that PCA can effectively discriminate between high- and low-risk patients, showing an explicit separation between the two groups. Similar to the PCA, the t-SNE plot revealed a distinct separation between both group patients, indicating the robustness of the risk score model in differentiating the two groups (Fig. 8B). Fig. 8C shows the PCA results for the four identified gene clusters. The plot shows distinct groupings for each cluster, highlighting the ability of the gene signature to effectively classify the IBD samples into separate clusters. Fig. 8D shows the t-SNE analysis of the four gene clusters. The t-SNE plot further confirmed the separation of the clusters identified by the PCA, with each cluster forming a distinct group. These dimensionality reduction analyses underscore the effectiveness of the gene signature in distinguishing between both group patients and in classifying IBD samples into distinct gene clusters, which can be crucial for understanding disease heterogeneity and tailoring personalized treatment strategies.

### 3.9 Associations between disease type, gene clusters, and risk groups

**Fig. 9:**
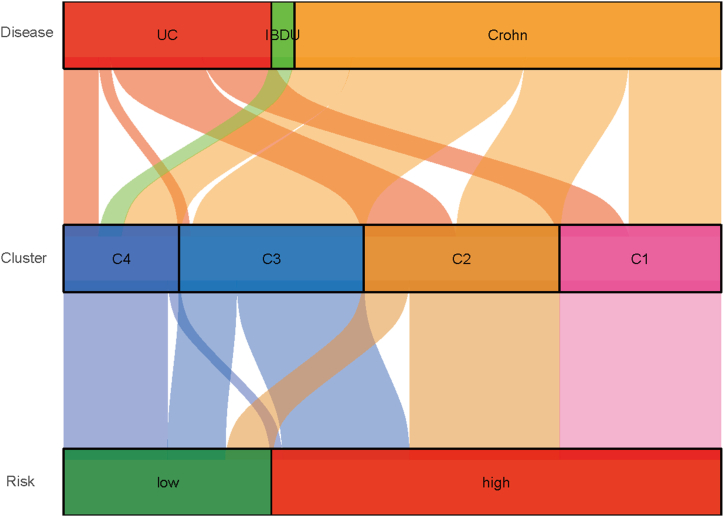
Sankey diagram. The Sankey diagram illustrates the relationships between disease type (UC, IBDU, and CD), gene clusters (C1, C2, C3, and C4), and risk groups (low, high). The flow lines represent the transitions of patients across these categories, with line thickness in proportion to the quantity of patients. The diagram highlights the heterogeneity within the population of patients with IBD and the associations between different levels.

According to Fig. 9, patients with ulcerative colitis (UC) are predominantly clustered into C1 and C2, with a significant number falling into the high-risk category. Patients with unclassified inflammatory bowel disease (IBDU) were mostly found in cluster C4 and tended to fall into the low-risk category. Patients with CD are distributed across all clusters, but showed a notable presence in cluster C3, with varying risk levels.

### 3.10 Expression levels of ERS degree-related genes and drug target marker genes

**Fig. 10:**
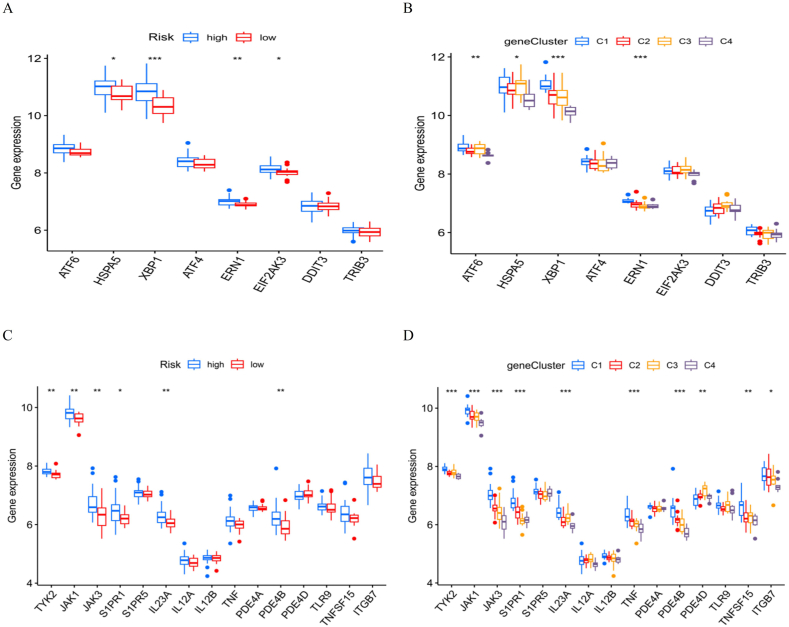
Expressions of ERS degree-associated genes and drug target marker genes. (A) Expressions of ERS-related genes (*ATF6, HSPA5, XBP1, ATF4, ERN1, EIF2AK3, DDIT3,* and *TRIB3*) between both groups. The plot indicates higher expression of several genes in the high-risk group. (B) Expression levels of ERS-related genes across the four gene clusters (C1, C2, C3, and C4). The variation in expression levels among clusters validated the classification based on ERS-related genes. (C) Expression levels of drug target marker genes (*TYK2, JAK1, JAK3, S1PR1, SIPR5, IL23A, IL12A, IL12B, TNF, PDE4A, PDE4B, PDE4D, TLR9, TNFSF15,* and *ITGB7*) between both groups. The high-risk group showed a significantly increased expression of these genes. (D) Expression levels of drug target marker genes across the four gene clusters. The discrepancies in expression modes among the clusters suggest varying responses to targeted therapies. *P* values are considered as not remarkable; *P < 0.05, **P < 0.01, and ***P < 0.001.

Fig. 10A shows the expression levels of ERS-related genes in high- and low-risk groups. The plot shows that half of the genes were significantly expressed in the high-risk group, indicating that our risk score model accurately reflected the degree of ERS in these patients. Specifically, genes such as *HSPA5, XBP1, ERN1*, and *EIF2AK3* were remarkably differentially expressed between both groups. Fig. 10B exhibits the expressions of ERS-related genes across four identified gene clusters (C1, C2, C3, and C4). The plot demonstrates that the expression levels varied among the clusters, further validating the classification based on ERS-related gene expression. This variation indicated that the gene clusters reflected distinct molecular profiles associated with ERS. Fig. 10C shows the expressions of the drug target marker genes in both groups. Consequently, it is shown that the expression levels of *TYK2, JAK1, JAK3, S1PR1, IL23A,* and *PDE4B* rose prominently in the high-risk group, which illustrated that these patients may have a heightened reaction to therapies targeting these markers. Fig. 10D shows the expressions of drug target marker genes in four gene clusters. The plot reveals disparities in the expression patterns of these genes in clusters, indicating that gene clusters can predict the efficacy of targeted drugs in different subgroups of patients with IBD. For instance, clusters C1 and C2 showed higher expression levels of several drug target genes than clusters C3 and C4. These analyses underscore the utility of the risk score model and gene clustering for identifying ERS-related molecular profiles and potential therapeutic targets, thereby expanding our comprehension of disease mechanisms and aiding the progress of personalized treatment strategies.

### 3.11 Immune cell levels and functions in high-risk and low-risk groups

**Fig. 11:**
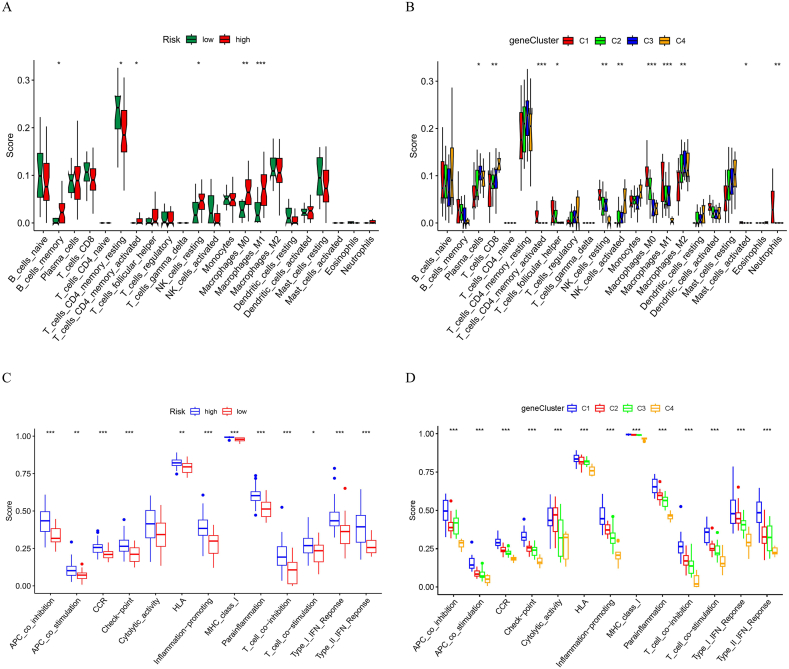
Immune cell levels and functions. (A) Comparison of immune cell levels between high-risk (red) and low-risk (green) groups. The high-risk group shows significantly more elevated levels of B cells memory, T cells CD4 memory activated, NK cell resting, macrophage M0, and macrophage M1, whereas T cells CD4 memory resting are lower. (B) Immune cell levels across four gene clusters (C1, C2, C3, and C4). Cluster 1 (red) shows a more elevated degree of immune infiltration. (C) Contrast of immune functions between high-risk (blue) and low-risk (red) groups. The high-risk group has significantly higher scores in most immune functions. (D) Immune functions across four gene clusters. Cluster 1 (blue) has higher scores in most immune functions, indicating a more active immune environment. *P* values are considered as not significant; *P < 0.05; **P < 0.01; and ***P < 0.001. HLA, human leukocyte antigen; APC, antigen-presenting cell; MHC, main histocompatibility complex; CCR, C-C chemokine receptor.

By comparing the number of immune cells in the high- and low-risk groups, we examined prominent distinctions in immune cell composition and function, highlighting the distinct immune landscapes of these groups. Fig. 11A illustrates the levels of diverse immune cells in both groups. The high-risk group exhibited significantly higher levels of B cell memory, T cell CD4 memory activation, NK cell resting, macrophage M0, and macrophage M1 than the low-risk group. Conversely, the levels of CD4 + memory resting are far lower in the high-risk group. Our findings showed a more elevated level of immune infiltration in the high-risk group. Fig. 11B shows the levels of immune cells across the four gene clusters (C1, C2, C3, and C4). Cluster 1 exhibited a more elevated degree of immune infiltration, with elevated levels of various immune cells, compared to other clusters. Fig. 11C shows the immune function of the high- and low-risk groups. Most immune functions, including APC costimulation, CCR, checkpoint, cytolytic activity, HLA, inflammation promotion, MHC class I, para-inflammation, T cell coinhibition, T cell costimulation, Type I IFN response, and Type II IFN response, were significantly higher in the high-risk group. This further supports the observation that the high-risk group experienced a more active immune environment. Fig. 11D shows the immune functions of the four gene clusters. Cluster 1 (blue) showed higher scores for most immune functions, suggesting that this cluster is characterized by a higher degree of immune activity and infiltration than the other clusters. These results underscore the distinct immune profiles of high-vs. low-risk groups and four gene clusters, providing insights into the immune mechanisms underlying the different risk categories and molecular subtypes of IBD.

### 3.12 Single-Cell Portal Analysis

**Fig. 12:**
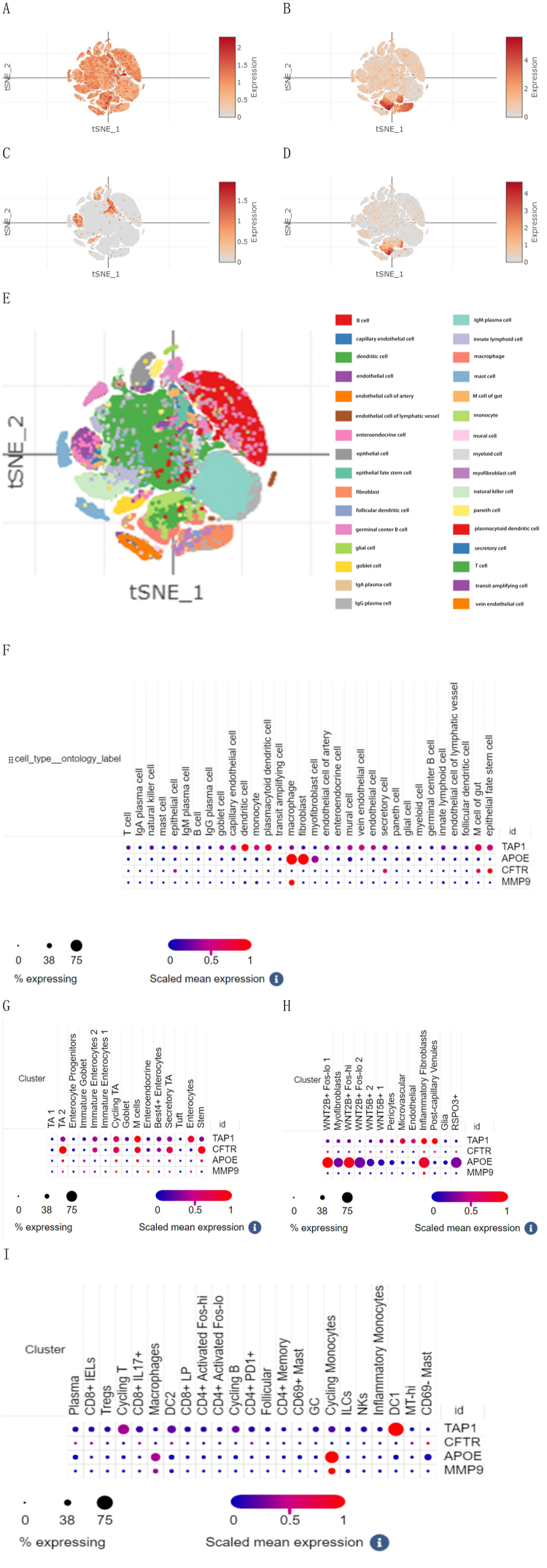
Analysis in single-cell sequence data. T-SNE plot of *TAP1* (A), *APOE* (B), *CFTR* (C), *MMP9* (D), and cell types (E) in CD. (F) Dot plot of cell subsets in CD. Dot plot of epithelial (G), stromal (H), and immune cell (I) subsets in UC.

In CD, *TAP1* was distributed in a variety of cells (Fig. 12A) and *APOE* was primarily distributed in macrophages, fibroblasts, and myofibroblasts (Fig. 12B). *CFTR* were primarily distributed in secretory cells, gut M cells, and epithelial stem cells (Fig. 12C). *MMP9* was primarily distributed in macrophages (Fig. 12D). Fig. 12E shows the cell types in the CD. Fig. 12F shows the degree of expression of the four ERS-related MGs in the 32 cell types. A similar distribution of cell subsets was observed in UC for the four MGs (Fig. 12G, H, and I).

## 4 Discussion

In addition to serving as a receptor for gut microbiota antigens and triggering intestinal inflammation, ERS is crucial for the maintenance of gastrointestinal homeostasis. When ERS is sufficiently severe, apoptotic pathways are activated, which work in tandem with ERS to compromise the intestinal mucosal barrier and promote IBD development. Understanding the mechanisms underlying ERS-related inflammation is essential for understanding the pathogenesis of IBD. ERS becomes an important factor regarding IBD susceptibility to IBD. Moreover, rebuilding intestinal homeostasis by rectifying ERS-associated signaling networks emerges as a possible treatment target for IBD [7]. We identified immune cells and pertinent hub genes exploring the ERS function in IBD and offering a novel standpoint on its etiology and therapy.

Forty-two ERS-associated DEGs were identified through differential gene expression analysis, 42 ERS-related DEGs were obtained. Enrichment analysis manifested that the genes chiefly got involved in ERS-associated inflammatory pathways, like the TNF- and Toll-like receptor signaling pathways. This finding suggests that IBD is closely associated with ERS. Subsequently, we identified four ERS-related MGs, namely: cystic fibrosis transmembrane conductance regulator *(CFTR),* transporter associated with antigen processing 1 *(TAP1),* matrix metallopeptidase 9 (*MMP-9*)*,* and apolipoprotein E (*APOE*).

*CFTR,* a chloride channel, also regulates sodium channel and bicarbonate transport in epithelial cells [10]. The ERS-related risk score predictive model showed that *CFTR* is a protective gene against IBD. According to previous studies, *CFTR* expression is significantly downregulated among active UC patients. Low *CFTR* gene expression substantially correlated with the clinical course [11], possibly because the loss of *CFTR* impacts the pH balance in colons. This leads to the pathogenic bacteria enrichment and the concomitant loss of organisms that can protect the mucosal surface [12]. Prior research has shown that the endogenous *CFTR* expression is significantly downregulated under ER stress [13]. Given the pathological conditions that cause ERS such as persistent inflammation in IBD, understanding *CFTR* expression regulation under these conditions is crucial.

*TAP1* encodes a protein required for the transfer of antigenic peptides across the ER membrane. Previous studies have shown that in affected IBD tissues, *TAP1* is overexpressed, and a positive immunostaining reaction is observed in the cytoplasm of epithelial cells [14]. This finding corresponds to the outcomes of the Single-Cell Portal Analysis of epithelial cells. *TAP1* becomes a strong tumor prognostic marker that has demonstrated clear prognostic relevance in most cancer types and is also an emerging predictor of clinic prognosis and immunotherapy response among various cancers [15]. However, only some studies have focused on *TAP1* expression among IBD patients. This is the first to link *TAP1*'s diagnostic value of with IBD. In terms of the control group, the ROC curve uncovered that *TAP1* with the maximal diagnostic value (AUC = 0.941) indicated the strong predictive value of *TAP1* for IBD risk, making it a promising potential predictor. Future longitudinal research needs performing to monitor the *TAP1* expression over time in patients with IBD, which will help determine whether changes in *TAP1* expression correlate with disease progression, treatment response, and relapse. In addition, researchers could explore the therapeutic potential of targeting *TAP1* in IBD by developing inhibitors or modulators of this gene and assessing its efficacy in preclinical and clinical settings.

*MMP-9*, a gelatinase derived from colonic epithelial cells and macrophages, is primarily involved in type IV collagen decomposition in the extracellular matrix. *MMP-9* as a pivotal adjustor of tissue damage in IBD plays a key role in its pathogenesis. ERS boosts FOS transcription, which triggers *MMP9* expression. Under pro-inflammatory conditions, ERS results in the additional induction of *MMP9* [16], making it a potential therapeutic target for IBD [17]. GS-5745 (andecaliximab), a humanized anti-*MMP9* monoclonal antibody, has been used in a clinical trial of IBD [18].

*APOE* is implicated in cholesterol, triglyceride, and other lipid transports and metabolic processes. As previously reported, *APOE* polymorphisms relate to an increased hazard of cultivating IBD and an earlier occurrence [19]. On account of its structural properties, *APOEε4* might be considered by the ER as misfolded, which activates the ERS reaction [20]. Evidence suggests that ERS is an early characteristic of *APOE*ε4 pathogenicity [21]. *APOE* is a major regulator of immunity and is associated with the regulation of macrophage-assisted antigen presentation [22]. Our Single-Cell Portal Analysis also showed that *APOE* was abundantly distributed in macrophages, suggesting that ERS may increase the risk of IBD through *APOE* regulation of macrophage inflammatory responses.

ERS activates the unfolded protein response (UPR) comprised of three primary branches: *IRE1, PERK*, and *ATF6*. Each branch contains a transmembrane protein showing a damage sensor transmitting signals to the nucleus by means of a distinctive transcription element. Our study identified the DEGs related to these pathways. Specifically, the expression levels of *ATF6* and *EIF2AK3* (also known as *PERK*) were examined and are presented in Fig. 10A and B. Notably, *ATF6* and *EIF2AK3*, the primary players in the UPR pathway, exhibited differential expression.

*ATF6* becomes a transcription factor activated upon ERS and translocates to the nucleus to induce the ER chaperone gene expression, enhance protein folding ability, and decrease stress [23]. In the high-risk group, the *ATF6* expression was upregulated contrasted to that in the low-risk group, suggesting a heightened ERS reaction. Elevated *ATF6* activity is connected to inflammation and epithelial cell apoptosis, exacerbating disease severity in IBD [23,24].

Similarly, *EIF2AK3/PERK* phosphorylates eIF2α brings about a decline in general protein synthesis, which alleviates ERS by reducing the influx of novel proteins into the ER. This pathway also promotes the stress-associated gene expressions, like ATF4 [25]. In the high-risk group, the *EIF2AK3* expression was prominently elevated, indicating its involvement in ER stress signaling and its potential role in exacerbating IBD. Dysregulation of *PERK* signaling can result in uncontrolled inflammation and cell death, contributing to the pathogenesis and progression of the disease [26,27].

Fig. 10B displays the expressions of the two genes across different gene groups, with significant variations observed in *ATF6*, emphasizing the heterogeneity of ER stress responses in patients with IBD. These findings highlighted the critical roles of these genes in the UPR pathway and their potential impact on IBD pathogenesis.

To explore the abnormal regulation of inflammatory cells in IBD, our research implemented the immune cell infiltration analysis. Consequently, it was shown that a prominent dysregulation of immune cells emerged in the colon tissues of IBD patients, with stronger immune functions in the high-risk group than those in the low-risk group. Positive relationships emerged among the risk score and neutrophils, M0 and M1 macrophages, T cells CD4 memory activated, and resting mast cells, with macrophages most closely correlated to the risk score. Single-Cell Portal Analysis revealed that all ERS-related MGs except *CFTR* were abundantly distributed in macrophages. Prior research has demonstrated that macrophages are important to intestinal homeostasis and immune defense while playing an important part in inhibiting intestinal inflammation [28]. ERS becomes an essential mediator of macrophage decomposition [29], and the *IRE1/XBP1* signaling pathway in ERS can mediate the inflammatory response of macrophages [30], suggesting that modulating ERS's impact of ERS on macrophages can provide a novel potential target for IBD therapy. In addition, blocking TLR signaling in macrophages may downregulate *CHOP* expression to alleviate ERS [31,32]. *C/EBP* or *CHOP* homologous protein is one transcription factor boosting apoptosis to respond to the long ERS [33]. Thus, regulating macrophages could ameliorate ERS and reduce the severity of IBD.

Fibrosis is a troublesome complication of IBD, featured by additional synthesis and deposition of the ECM and remodeling of the submucosal tissue [34]. The activation and transformation of fibroblasts into myofibroblasts are central to fibrogenesis. Myofibroblasts become the dominant effector cells in the progression of intestinal fibrosis. As inflammation becomes chronic, hyperactive intestinal myofibroblasts undergo excessive ECM synthesis and cytokine release, causing fibrosis. Single-Cell Portal Analysis showed that ERS-related MG *APOE* was abundantly distributed in fibroblasts and myofibroblasts. In addition, GSEA indicated that ERS-related DEGs were significantly enriched in ECM receptor interactions, linking ERS closely to myofibroblasts and fibrosis in IBD. Previous studies have found that ERS can activate fibroblasts and transform them into myofibroblasts [35], consistent with our finding that fibrosis in IBD can be alleviated by downregulating ERS.

Precision medicine aims to classify individuals with similar features into subgroups based on specific therapies. This study focused on a precise molecular therapy for specific groups with predictable outcomes. Currently, IBD treatment primarily involves phenotyping patients and administering non-specific anti-inflammatory drugs (such as 5-aminosalicylic acid and corticosteroids). However, these treatments are not adequately targeted, have significant side effects, and tolerance can easily develop tolerance [[36], [37], [38]]. Recent monoclonal antibody therapies targeting inflammatory factors such as TNF- and IL-12/IL-23 have shown success, but many individuals remain non-responsive [39]. Sensitive diagnostic markers for patients with IBD who fail to respond to therapy are urgently required. This study aimed to provide a molecular classification of IBD based on genomic research and targeted therapies for various molecules. A genetic risk score model composed of four ERS-related MGs was employed to rate the effectiveness of the targeted treatment, suggesting distinctions in target marker gene expression among patients with different risk scores and gene clusters. Our research puts forward a novel tactic for the accurate therapy of IBD, indicating that high-risk patients may respond better to targeted therapies. Most targets differed between gene clusters, highlighting the practical value of gene grouping for the accurate selection of targeted medicines.

## 5 Conclusions

Our research mainly aimed to recognize risk factors and high-risk groups at the molecular level for IBD. We successfully established a predictive model according to four key genes, namely *TAP1, MMP9, CFTR*, and *APOE*. This model effectively distinguished between high- and low-risk populations, and identified gene clusters with distinct treatment responses in IBD.

Having combined immune infiltration data and single-cell analysis, we regarded macrophages as the most related immune cell type concerning the IBD risk score. Our novel ERS-related gene model can predict IBD patient risk, immunological state, and treatment efficacy, offering valuable insights into personalized therapy.

In addition, our study highlighted the critical roles of the UPR and ERS pathways in IBD. Chronic ER stress activates the UPR to restore normal function; however, both in immune cells and intestinal epithelial, persistent ER stress exacerbates inflammation and tissue damage, contributing to IBD progression. Monitoring the UPR-ER pathway provides a potential avenue for managing IBD, emphasizing the significance of our ERS-related gene model for predicting patient outcomes and tailoring treatments.

## 6 Study limitations

Although this research offers significant perceptions on the classification and molecular characterization of patients with IBD using ERS-related genes, several limitations should be acknowledged.

(1) Heterogeneity of IBD: IBD is an exceedingly heterogeneous illness with varying clinic manifestations and genetic backgrounds. Our study focused on ERS-related genes; however, other molecular pathways and environmental factors also play critical roles in IBD pathogenesis and were not addressed in this study.
(2) Lack of Multi-omics Data: Although study utilized transcriptomics and single-cell RNA sequencing data, it lacked integration with other omics approaches, such as proteomics, metabolomics, and epigenomics. Future studies should incorporate these multi-omics data to obtain an integrated comprehension of IBD pathogenesis and improve the predictive power of the models.

## Ethics approval and consent to participate

Inapplicable.

## Data availability statement

The datasets are supplied by the Gene Expression Omnibus database (http://www.ncbi.nlm.nih.gov/geo/), including GSE126124 (platform: GPL6244) and GSE75214 (platform: GPL6244). The data supporting our research is supplied by the corresponding author on rational requirement.

## CRediT authorship contribution statement

**Ziyu Liu:** Writing – review & editing, Writing – original draft, Visualization, Validation, Project administration, Methodology, Investigation, Formal analysis, Data curation, Conceptualization. **Zahra Zeinalzadeh:** Writing – review & editing, Writing – original draft, Validation. **Tao Huang:** Writing – original draft. **Yingying Han:** Writing – original draft. **Lushan Peng:** Writing – original draft. **Dan Wang:** Writing – original draft. **Zongjiang Zhou:** Writing – original draft. **Diabate Ousmane:** Writing – original draft. **Junpu Wang:** Writing – review & editing, Supervision, Resources, Project administration, Methodology, Funding acquisition, Formal analysis, Conceptualization.

## Declaration of competing interest

The authors declare the following financial interests/personal relationships which may be considered as potential competing interests: Junpu Wang reports the financial support was provided by the 10.13039/501100001809National Natural Science Foundation of China (No. 81602167), the 10.13039/501100004735Hunan Provincial Natural Science Foundation of China (No. 2017JJ3494 and 2021JJ31100), and the Science and Technology Program Foundation of Changsha City (No. kq2004085).
