# Supplementary material for: Identification of endoplasmic reticulum stress-associated genes and subtypes for predicting risk signature and depicting immune features in inflammatory bowel disease
Source: Heliyon. 2024 Sep 1;10(17):e37053. doi: 10.1016/j.heliyon.2024.e37053 (PMC11409092; doi:10.1016/j.heliyon.2024.e37053)
Supplement: Multimedia component 1 [file mmc1.docx]

Supplementary Table 1. 502 differentially expressed genes from a total of 23306 genes

| id | logFC | AveExpr | t | P.Value | adj.P.Val | B |
| --- | --- | --- | --- | --- | --- | --- |
| *UBE2L6* | 0.773255838 | 8.038130186 | 8.990804023 | 9.21E-14 | 1.07E-09 | 20.8415549 |
| *HLA-F* | 0.763120384 | 9.9805502 | 8.618771652 | 4.96E-13 | 3.85E-09 | 19.23660993 |
| *PSMB9* | 1.082076852 | 9.666131358 | 8.529771744 | 7.42E-13 | 4.32E-09 | 18.85247105 |
| *FCN1* | 0.771400536 | 7.352945456 | 8.349068228 | 1.68E-12 | 7.83E-09 | 18.07261942 |
| *MDK* | 0.835361913 | 8.819606069 | 7.934080964 | 1.09E-11 | 3.10E-08 | 16.28408975 |
| *IRF1* | 0.968037012 | 8.944239898 | 7.930790743 | 1.11E-11 | 3.10E-08 | 16.26993268 |
| *TYMP* | 0.750023663 | 8.032599067 | 7.845746579 | 1.63E-11 | 3.16E-08 | 15.90418192 |
| *SLC23A3* | -0.787892195 | 6.508829049 | -7.452026662 | 9.53E-11 | 9.85E-08 | 14.21651374 |
| *PSMB10* | 0.672727058 | 7.974096202 | 7.409434034 | 1.15E-10 | 9.85E-08 | 14.03463048 |
| *FLVCR1* | -0.627065061 | 6.469981579 | -7.407966996 | 1.16E-10 | 9.85E-08 | 14.02836859 |
| *PDZK1IP1* | 1.640206504 | 9.134608563 | 7.397379658 | 1.22E-10 | 9.85E-08 | 13.98318339 |
| *CYP4F2* | -0.85867332 | 5.705550518 | -7.355359309 | 1.47E-10 | 1.09E-07 | 13.80394634 |
| *IFI35* | 0.65073073 | 8.445830342 | 7.330827927 | 1.64E-10 | 1.13E-07 | 13.69938364 |
| *CFB* | 1.516284804 | 8.808494444 | 7.32070857 | 1.71E-10 | 1.13E-07 | 13.65626745 |
| *PRKG2* | -1.47312707 | 5.05444164 | -7.320655366 | 1.71E-10 | 1.13E-07 | 13.65604079 |
| *RARRES3* | 1.130327867 | 8.235142392 | 7.309387805 | 1.80E-10 | 1.13E-07 | 13.60804403 |
| *LCN2* | 2.002045055 | 9.92125083 | 7.174981884 | 3.28E-10 | 1.82E-07 | 13.03650058 |
| *SLC16A9* | -1.538086276 | 6.527235056 | -7.166513356 | 3.40E-10 | 1.82E-07 | 13.00055335 |
| *TAP1* | 0.839318495 | 9.612641669 | 7.159569451 | 3.51E-10 | 1.82E-07 | 12.97108377 |
| *APOL1* | 1.428963532 | 8.047132313 | 7.133747483 | 3.93E-10 | 1.95E-07 | 12.86154402 |
| *HLA-DMA* | 0.792067189 | 8.86644145 | 7.0975579 | 4.62E-10 | 2.07E-07 | 12.70815184 |
| *C1QB* | 0.742445429 | 9.093255944 | 7.032285604 | 6.17E-10 | 2.49E-07 | 12.43188246 |
| *TYROBP* | 0.839852193 | 9.248271587 | 7.010132453 | 6.81E-10 | 2.64E-07 | 12.33823633 |
| *FCGR3A* | 1.347749108 | 7.057836838 | 6.969290254 | 8.15E-10 | 3.00E-07 | 12.1657506 |
| *TAP2* | 0.72535692 | 7.894794186 | 6.957834364 | 8.58E-10 | 3.08E-07 | 12.11740853 |
| *CNTN4* | -0.728035621 | 6.09718769 | -6.943005892 | 9.16E-10 | 3.23E-07 | 12.05486031 |
| *ZC3H12A* | 0.857840815 | 8.143008317 | 6.928137212 | 9.78E-10 | 3.40E-07 | 11.99217182 |
| *SETD9* | -0.627271642 | 5.941164322 | -6.881833849 | 1.20E-09 | 3.99E-07 | 11.79714209 |
| *SPINK1* | 1.043415914 | 9.414951904 | 6.877555997 | 1.22E-09 | 4.01E-07 | 11.77913868 |
| *DUOX2* | 3.093904655 | 8.927416163 | 6.809276945 | 1.65E-09 | 5.24E-07 | 11.49213653 |
| *ADM* | 1.23318242 | 8.484223853 | 6.772458471 | 1.94E-09 | 5.80E-07 | 11.33765668 |
| *TGM2* | 1.322362947 | 8.477508976 | 6.743519157 | 2.20E-09 | 6.04E-07 | 11.21637863 |
| *CAPN13* | -0.903111354 | 5.755782996 | -6.739905837 | 2.24E-09 | 6.06E-07 | 11.20124502 |
| *BACE2* | 0.679219318 | 8.916459037 | 6.726641349 | 2.37E-09 | 6.17E-07 | 11.14570688 |
| *C2* | 1.022265995 | 7.967031879 | 6.665704502 | 3.10E-09 | 7.64E-07 | 10.89092115 |
| *CYP2C9* | -0.752706387 | 4.964935579 | -6.6396989 | 3.47E-09 | 8.34E-07 | 10.7823699 |
| *DPP10* | -0.845426706 | 5.187925873 | -6.61236782 | 3.91E-09 | 9.12E-07 | 10.66840624 |
| *UGDH* | -0.71144448 | 10.90224387 | -6.606236618 | 4.02E-09 | 9.26E-07 | 10.64285784 |
| *PAQR5* | -0.873227635 | 7.372188639 | -6.604345039 | 4.05E-09 | 9.26E-07 | 10.63497701 |
| *CFI* | 1.240732704 | 6.748125433 | 6.541952753 | 5.32E-09 | 1.12E-06 | 10.37537713 |
| *TNFRSF1B* | 0.624368428 | 7.644134917 | 6.536988055 | 5.43E-09 | 1.13E-06 | 10.35474922 |
| *ABCB1* | -1.267165576 | 7.725336028 | -6.44944648 | 7.95E-09 | 1.47E-06 | 9.991748446 |
| *ASS1* | 0.760773038 | 10.87225467 | 6.449041734 | 7.96E-09 | 1.47E-06 | 9.990073382 |
| *CYP4X1* | 0.596910463 | 5.22353556 | 6.403796523 | 9.69E-09 | 1.65E-06 | 9.803017825 |
| *C1QA* | 0.713101566 | 7.748225044 | 6.358722912 | 1.18E-08 | 1.86E-06 | 9.617061945 |
| *LILRB2* | 0.59065588 | 6.929692463 | 6.345934609 | 1.24E-08 | 1.92E-06 | 9.564374881 |
| *IFITM1* | 0.727391057 | 10.34439086 | 6.336712661 | 1.29E-08 | 1.95E-06 | 9.526401119 |
| *CXCL1* | 1.657893519 | 8.724111483 | 6.306640334 | 1.47E-08 | 2.09E-06 | 9.402688923 |
| *STAT1* | 0.987271979 | 10.1384179 | 6.303597784 | 1.49E-08 | 2.11E-06 | 9.390182594 |
| *PLA1A* | 0.65478853 | 5.486087847 | 6.265325051 | 1.76E-08 | 2.37E-06 | 9.233025431 |
| *MZB1* | 0.995503929 | 8.567944058 | 6.248539774 | 1.89E-08 | 2.49E-06 | 9.164196737 |
| *QSOX1* | 0.67396828 | 8.790976682 | 6.230482049 | 2.05E-08 | 2.56E-06 | 9.090216428 |
| *S100A8* | 2.391658594 | 7.199895063 | 6.221575139 | 2.13E-08 | 2.59E-06 | 9.053751344 |
| *UGT1A1* | -1.083821549 | 7.143326554 | -6.154757157 | 2.83E-08 | 3.23E-06 | 8.780742686 |
| *C5orf63* | -0.641312927 | 7.04082668 | -6.149008514 | 2.90E-08 | 3.24E-06 | 8.757300236 |
| *SLC40A1* | 0.642291871 | 10.38693788 | 6.147719251 | 2.92E-08 | 3.24E-06 | 8.752043744 |
| *SLC19A3* | -0.807032949 | 6.404254727 | -6.133152456 | 3.10E-08 | 3.35E-06 | 8.692678773 |
| *IFI30* | 0.771703668 | 10.71159605 | 6.125187041 | 3.21E-08 | 3.42E-06 | 8.660236913 |
| *DERL3* | 0.686711936 | 7.453970959 | 6.100030882 | 3.58E-08 | 3.74E-06 | 8.557873972 |
| *REG1A* | 2.907787714 | 11.13200084 | 6.068919714 | 4.08E-08 | 4.18E-06 | 8.43147948 |
| *PROK2* | 0.946083437 | 5.947894418 | 6.066173855 | 4.13E-08 | 4.18E-06 | 8.420334689 |
| *STRADB* | -0.644683449 | 8.141876656 | -6.065310605 | 4.15E-08 | 4.18E-06 | 8.416831322 |
| *CYP2B7P* | -1.211691262 | 5.623070409 | -6.063960113 | 4.17E-08 | 4.18E-06 | 8.411350909 |
| *FCGR1A* | 1.283860267 | 5.945698404 | 6.063601169 | 4.18E-08 | 4.18E-06 | 8.409894355 |
| *GLB1L2* | -0.687585508 | 7.603666318 | -6.06121486 | 4.22E-08 | 4.18E-06 | 8.400211745 |
| *PI3* | 2.205426837 | 8.719281929 | 6.055008312 | 4.33E-08 | 4.18E-06 | 8.37503447 |
| *SLC38A4* | -1.676005958 | 6.343453625 | -6.04898222 | 4.44E-08 | 4.18E-06 | 8.35059785 |
| *DUOXA2* | 2.225169237 | 7.964320697 | 6.042377348 | 4.57E-08 | 4.23E-06 | 8.323823983 |
| *IGKV1OR9-2* | 0.596615803 | 8.931638609 | 6.041823175 | 4.58E-08 | 4.23E-06 | 8.321578024 |
| *MUC1* | 0.806977477 | 9.832653509 | 6.041513283 | 4.59E-08 | 4.23E-06 | 8.320322123 |
| *HLA-DRB3* | 0.788644665 | 11.1148858 | 5.99575044 | 5.57E-08 | 4.94E-06 | 8.135108888 |
| *ZNF91* | -0.777107052 | 7.735927767 | -5.992796975 | 5.64E-08 | 4.98E-06 | 8.123172736 |
| *IGKV2D-28* | 0.872515598 | 12.27731067 | 5.982888449 | 5.88E-08 | 5.13E-06 | 8.083143779 |
| *AHCYL2* | -0.777088842 | 10.31473577 | -5.980211134 | 5.95E-08 | 5.16E-06 | 8.072331912 |
| *TTLL6* | -0.714729872 | 5.813519369 | -5.976006583 | 6.06E-08 | 5.17E-06 | 8.055356101 |
| *HOXA2* | -0.67494567 | 6.387549658 | -5.9647403 | 6.35E-08 | 5.30E-06 | 8.009889918 |
| *KCND3* | 0.765793781 | 6.107869319 | 5.952624566 | 6.69E-08 | 5.43E-06 | 7.961030439 |
| *C7orf31* | -0.607638705 | 6.544893942 | -5.943660493 | 6.95E-08 | 5.58E-06 | 7.924904062 |
| *GBP4* | 1.175198123 | 7.779374382 | 5.939696804 | 7.07E-08 | 5.60E-06 | 7.908936232 |
| *RMDN2* | -0.764622355 | 6.513030185 | -5.931219234 | 7.32E-08 | 5.69E-06 | 7.874797216 |
| *HLA-DRB1* | 0.748595123 | 10.90839274 | 5.925548062 | 7.50E-08 | 5.80E-06 | 7.851969518 |
| *STX11* | 0.640808547 | 6.671947041 | 5.917766372 | 7.75E-08 | 5.96E-06 | 7.820659637 |
| *TGFBI* | 0.686988463 | 8.985713066 | 5.914946662 | 7.84E-08 | 6.01E-06 | 7.809318197 |
| *RAB31* | 0.970661893 | 8.315422121 | 5.909316793 | 8.03E-08 | 6.10E-06 | 7.786679715 |
| *PPP2R3A* | -0.655936837 | 6.523348291 | -5.901004802 | 8.32E-08 | 6.22E-06 | 7.753270684 |
| *ITGB2* | 0.758598348 | 8.337267288 | 5.892318325 | 8.63E-08 | 6.29E-06 | 7.718375166 |
| *TSPAN7* | -0.834166788 | 9.948180802 | -5.874522516 | 9.30E-08 | 6.65E-06 | 7.646945507 |
| *SLC4A10* | -0.934360654 | 5.758014198 | -5.8693974 | 9.51E-08 | 6.73E-06 | 7.62638913 |
| *ZBP1* | 0.65399565 | 7.097406724 | 5.862763693 | 9.78E-08 | 6.88E-06 | 7.599791983 |
| *GBP2* | 0.871691052 | 7.522632575 | 5.857201315 | 1.00E-07 | 7.02E-06 | 7.577498977 |
| *IGSF6* | 0.859115616 | 7.548829834 | 5.854333834 | 1.01E-07 | 7.09E-06 | 7.56600976 |
| *SMOC2* | 0.673081097 | 6.807545143 | 5.829751217 | 1.12E-07 | 7.42E-06 | 7.467601795 |
| *IGFBP7* | 0.759653006 | 8.83283604 | 5.809176484 | 1.22E-07 | 7.93E-06 | 7.385359972 |
| *CD38* | 0.828852858 | 7.693305213 | 5.804266056 | 1.25E-07 | 8.07E-06 | 7.365748457 |
| *IGKV1D-27* | 1.174574003 | 9.723870108 | 5.797820353 | 1.28E-07 | 8.21E-06 | 7.340015025 |
| *CYP2C19* | -0.646749294 | 5.752279962 | -5.796302448 | 1.29E-07 | 8.21E-06 | 7.333956645 |
| *ABCG2* | -1.946086785 | 7.454206797 | -5.796159734 | 1.29E-07 | 8.21E-06 | 7.333387066 |
| *WARS* | 0.998641352 | 9.234051802 | 5.793289144 | 1.31E-07 | 8.22E-06 | 7.321931535 |
| *APOE* | 0.63592186 | 8.523854345 | 5.778281918 | 1.39E-07 | 8.64E-06 | 7.262078863 |
| *SLC39A2* | -0.641666076 | 5.230397611 | -5.776289208 | 1.41E-07 | 8.64E-06 | 7.254135983 |
| *LOC102724453* | 0.853993556 | 12.16331367 | 5.771045369 | 1.44E-07 | 8.77E-06 | 7.233239312 |
| *SDR42E1* | -0.612858147 | 6.41334637 | -5.765577012 | 1.47E-07 | 8.83E-06 | 7.211455864 |
| *FMO4* | -0.74578178 | 6.869169127 | -5.7565594 | 1.53E-07 | 9.01E-06 | 7.175551522 |
| *LGALS1* | 0.803985992 | 9.110269489 | 5.756128278 | 1.53E-07 | 9.01E-06 | 7.173835529 |
| *CD82* | 0.673987605 | 8.354116888 | 5.742858312 | 1.62E-07 | 9.42E-06 | 7.121041972 |
| *NOS2* | 1.904165723 | 8.584904795 | 5.726186097 | 1.73E-07 | 9.75E-06 | 7.05478131 |
| *AQP9* | 1.901530576 | 5.964653814 | 5.724710434 | 1.74E-07 | 9.75E-06 | 7.048920248 |
| *NTRK2* | -0.622220489 | 5.422656907 | -5.720690295 | 1.77E-07 | 9.75E-06 | 7.03295606 |
| *SLC25A33* | -0.586342084 | 6.970288444 | -5.708994345 | 1.86E-07 | 1.01E-05 | 6.98653632 |
| *PLA2G16* | 0.632533102 | 7.15815433 | 5.702892207 | 1.91E-07 | 1.03E-05 | 6.962332833 |
| *HCST* | 0.626195973 | 7.491482272 | 5.701957722 | 1.92E-07 | 1.03E-05 | 6.958627213 |
| *REG1B* | 3.510869555 | 8.546026482 | 5.686689394 | 2.04E-07 | 1.08E-05 | 6.898116683 |
| *IGKV2D-18* | 0.723033639 | 7.196422159 | 5.68299149 | 2.08E-07 | 1.09E-05 | 6.883471228 |
| *CYP2C18* | -0.891400478 | 7.771250375 | -5.656296697 | 2.32E-07 | 1.18E-05 | 6.777862116 |
| *SLPI* | 1.056727645 | 8.555148067 | 5.653996483 | 2.34E-07 | 1.18E-05 | 6.768771576 |
| *SOD2* | 0.769709986 | 9.800841768 | 5.65378996 | 2.34E-07 | 1.18E-05 | 6.767955462 |
| *RN7SL564P* | 0.643357893 | 9.618937385 | 5.650710925 | 2.37E-07 | 1.19E-05 | 6.755789545 |
| *DMBT1* | 2.237026651 | 10.39970682 | 5.634537054 | 2.54E-07 | 1.24E-05 | 6.691927869 |
| *PTGR1* | -0.594655318 | 8.489054028 | -5.633758705 | 2.55E-07 | 1.24E-05 | 6.6888565 |
| *C1R* | 0.770157273 | 9.097965331 | 5.628645693 | 2.60E-07 | 1.26E-05 | 6.668684884 |
| *IGKV1D-33* | 0.642170507 | 11.42424693 | 5.624724964 | 2.64E-07 | 1.28E-05 | 6.653222133 |
| *IGKV1D-42* | 0.719264664 | 12.83480837 | 5.622303678 | 2.67E-07 | 1.29E-05 | 6.643675182 |
| *TRHDE* | -0.861804766 | 5.443050703 | -5.607719608 | 2.84E-07 | 1.35E-05 | 6.586207368 |
| *TWSG1* | 0.605781685 | 7.529361619 | 5.583737382 | 3.13E-07 | 1.44E-05 | 6.491842021 |
| *IFITM3* | 0.672223416 | 9.047189593 | 5.554645318 | 3.53E-07 | 1.58E-05 | 6.377598974 |
| *LINC00152* | 0.64154275 | 8.161289336 | 5.544159263 | 3.69E-07 | 1.63E-05 | 6.336482798 |
| *DYSF* | 0.65749381 | 5.691105421 | 5.533614152 | 3.85E-07 | 1.69E-05 | 6.295168457 |
| *ZG16B* | 0.749884793 | 7.801929551 | 5.524362255 | 4.00E-07 | 1.74E-05 | 6.258948469 |
| *GCNT2* | -0.843346896 | 6.149332353 | -5.520976311 | 4.06E-07 | 1.76E-05 | 6.24569943 |
| *LILRA3* | 0.624876078 | 5.62417369 | 5.518644408 | 4.10E-07 | 1.77E-05 | 6.236576834 |
| *PLCE1* | -0.853761628 | 8.755483727 | -5.514019045 | 4.18E-07 | 1.78E-05 | 6.218486937 |
| *PPARGC1A* | -0.856108653 | 7.486743638 | -5.498901993 | 4.45E-07 | 1.86E-05 | 6.15940945 |
| *CNTN3* | -0.706167782 | 5.953395139 | -5.497172003 | 4.48E-07 | 1.87E-05 | 6.152653119 |
| *ABCA8* | -0.909971031 | 5.758841574 | -5.491457515 | 4.58E-07 | 1.89E-05 | 6.130342203 |
| *LYN* | 0.654319246 | 8.512019231 | 5.485188335 | 4.70E-07 | 1.92E-05 | 6.105877205 |
| *SLC16A1* | -0.873703656 | 9.341199603 | -5.483520046 | 4.74E-07 | 1.92E-05 | 6.099368883 |
| *NXPE2* | -0.85376109 | 6.925261684 | -5.481455666 | 4.78E-07 | 1.93E-05 | 6.091316526 |
| *FSIP2* | -0.69744458 | 5.662124225 | -5.478822669 | 4.83E-07 | 1.93E-05 | 6.081048123 |
| *PDE8A* | -0.586972227 | 8.375577588 | -5.471486243 | 4.98E-07 | 1.98E-05 | 6.052448222 |
| *ASS1P11* | 0.673541834 | 6.632917567 | 5.463632827 | 5.14E-07 | 2.02E-05 | 6.021851482 |
| *SLC22A5* | -0.886387052 | 8.404549343 | -5.462676984 | 5.16E-07 | 2.02E-05 | 6.018128852 |
| *MEP1B* | -1.765439073 | 7.62747476 | -5.457355028 | 5.27E-07 | 2.06E-05 | 5.997407165 |
| *CCL3* | 0.876603614 | 6.929165494 | 5.449567235 | 5.44E-07 | 2.10E-05 | 5.967100443 |
| *XBP1* | 0.607477573 | 10.48800185 | 5.43158746 | 5.86E-07 | 2.21E-05 | 5.89720386 |
| *RNA5SP443* | 0.921356499 | 6.456479617 | 5.422393766 | 6.08E-07 | 2.27E-05 | 5.861502805 |
| *VNN1* | 1.732484212 | 6.318782497 | 5.421635066 | 6.10E-07 | 2.27E-05 | 5.858557812 |
| *RNA5SP382* | 0.638308149 | 9.928791852 | 5.418134839 | 6.19E-07 | 2.30E-05 | 5.844973599 |
| *SUGCT* | -0.6319099 | 6.264055052 | -5.402977791 | 6.59E-07 | 2.40E-05 | 5.786194978 |
| *COL6A1* | 0.642314152 | 8.300134034 | 5.394143904 | 6.83E-07 | 2.46E-05 | 5.751971384 |
| *WDR78* | -0.723721528 | 6.560546569 | -5.384017869 | 7.11E-07 | 2.51E-05 | 5.712772777 |
| *SLC35G1* | -0.653825104 | 7.223626637 | -5.372480424 | 7.46E-07 | 2.58E-05 | 5.668150954 |
| *PDZD3* | -0.759401404 | 6.933613589 | -5.368103076 | 7.59E-07 | 2.61E-05 | 5.651232589 |
| *SLC10A5* | -0.921873856 | 5.282359208 | -5.362596573 | 7.76E-07 | 2.65E-05 | 5.629958937 |
| *RNU5A-8P* | 0.977885196 | 5.794499984 | 5.336828437 | 8.62E-07 | 2.86E-05 | 5.530539133 |
| *IDO1* | 1.61508251 | 7.492656819 | 5.318311017 | 9.29E-07 | 3.00E-05 | 5.459229689 |
| *CTGF* | 0.712576907 | 7.818746846 | 5.315537114 | 9.40E-07 | 3.01E-05 | 5.448557372 |
| *LYZ* | 0.690860648 | 11.38516849 | 5.310237605 | 9.60E-07 | 3.05E-05 | 5.428175167 |
| *CXCL11* | 1.170498681 | 5.705181159 | 5.298985471 | 1.01E-06 | 3.13E-05 | 5.38492998 |
| *CASP1* | 0.669035532 | 9.172069118 | 5.291226109 | 1.04E-06 | 3.18E-05 | 5.355133278 |
| *IGKC* | 0.595556207 | 11.67579825 | 5.287443457 | 1.05E-06 | 3.22E-05 | 5.340614878 |
| *SGK2* | -0.868275183 | 7.969420649 | -5.281388318 | 1.08E-06 | 3.27E-05 | 5.317384374 |
| *GXYLT2* | -0.70054234 | 6.894987626 | -5.280208518 | 1.08E-06 | 3.27E-05 | 5.312859522 |
| *RUNDC3B* | -0.589450133 | 5.868056959 | -5.269120052 | 1.13E-06 | 3.37E-05 | 5.270355309 |
| *OTC* | -0.822929128 | 5.710032723 | -5.268830091 | 1.14E-06 | 3.37E-05 | 5.269244394 |
| *AKR1B10* | -0.909971379 | 8.586564051 | -5.268359912 | 1.14E-06 | 3.37E-05 | 5.267443078 |
| *HLA-DPB1* | 0.632438213 | 10.59558061 | 5.263441444 | 1.16E-06 | 3.43E-05 | 5.248604298 |
| *GPR125* | -0.589038773 | 7.971962642 | -5.255008896 | 1.20E-06 | 3.51E-05 | 5.216325041 |
| *SLC20A1* | -0.960512368 | 9.246917145 | -5.250630761 | 1.22E-06 | 3.55E-05 | 5.199575408 |
| *CHI3L1* | 1.957405114 | 7.514981385 | 5.242157791 | 1.26E-06 | 3.64E-05 | 5.167178649 |
| *IGKV2D-24* | 1.003331154 | 10.17164583 | 5.241234429 | 1.27E-06 | 3.65E-05 | 5.163649627 |
| *CYP4F12* | -0.938698638 | 6.82226356 | -5.233029504 | 1.31E-06 | 3.74E-05 | 5.132303893 |
| *RNA5SP276* | 0.671094321 | 5.132014317 | 5.223204761 | 1.36E-06 | 3.85E-05 | 5.094800473 |
| *CROT* | -0.590790191 | 7.586261396 | -5.221695632 | 1.37E-06 | 3.87E-05 | 5.089042723 |
| *IGKV1D-43* | 0.642435187 | 11.04704728 | 5.218050809 | 1.39E-06 | 3.90E-05 | 5.075139966 |
| *PIM2* | 0.974128699 | 8.695464111 | 5.203146666 | 1.48E-06 | 4.07E-05 | 5.018337934 |
| *PRDM1* | 0.716772693 | 8.424791344 | 5.198996484 | 1.50E-06 | 4.12E-05 | 5.002534745 |
| *ARFGAP3* | 0.625985624 | 7.713117767 | 5.198451268 | 1.51E-06 | 4.12E-05 | 5.000459102 |
| *DRAM1* | 0.779208957 | 7.051859973 | 5.195687965 | 1.52E-06 | 4.16E-05 | 4.989940786 |
| *ADH6* | -0.617533495 | 6.581852372 | -5.188887449 | 1.57E-06 | 4.24E-05 | 4.964066519 |
| *NBEAL1* | -0.606266465 | 7.983202999 | -5.181222347 | 1.62E-06 | 4.31E-05 | 4.934922222 |
| *OLFM4* | 0.864625622 | 11.76372704 | 5.17470008 | 1.66E-06 | 4.37E-05 | 4.910139541 |
| *ELL2* | 0.605265143 | 8.101652093 | 5.167802402 | 1.71E-06 | 4.45E-05 | 4.883946786 |
| *IGKV1OR2-3* | 0.834488002 | 10.80147133 | 5.151727282 | 1.82E-06 | 4.67E-05 | 4.822969868 |
| *MYO1D* | -0.59793898 | 10.19570679 | -5.144962925 | 1.87E-06 | 4.75E-05 | 4.797338541 |
| *PLA2R1* | -0.587972029 | 6.398439476 | -5.143535526 | 1.88E-06 | 4.77E-05 | 4.791931971 |
| *SULT1B1* | -0.617832327 | 10.49302374 | -5.142591544 | 1.89E-06 | 4.78E-05 | 4.788356844 |
| *SCD* | 0.705257444 | 8.316825345 | 5.140725656 | 1.90E-06 | 4.81E-05 | 4.781291141 |
| *FSTL1* | 0.858629421 | 8.887690536 | 5.135533466 | 1.94E-06 | 4.83E-05 | 4.761636041 |
| *CCL18* | 1.620163552 | 7.803307716 | 5.134273468 | 1.95E-06 | 4.84E-05 | 4.756867763 |
| *APOL2* | 0.611002635 | 7.205882044 | 5.119935122 | 2.06E-06 | 5.06E-05 | 4.702646705 |
| *GBP5* | 1.260020869 | 7.312055802 | 5.114032592 | 2.11E-06 | 5.12E-05 | 4.680347635 |
| *FADS1* | 0.751222654 | 6.361517101 | 5.11346008 | 2.12E-06 | 5.12E-05 | 4.678185422 |
| *RNU2-2P* | 0.934816148 | 8.850958768 | 5.107678455 | 2.17E-06 | 5.20E-05 | 4.656356569 |
| *RNA5SP19* | 1.022906167 | 7.678857195 | 5.099494777 | 2.24E-06 | 5.32E-05 | 4.625479421 |
| *SCUBE2* | -0.621023909 | 6.43193723 | -5.097792469 | 2.26E-06 | 5.34E-05 | 4.619059657 |
| *LOC100508226* | -0.595036252 | 5.441088928 | -5.094194294 | 2.29E-06 | 5.38E-05 | 4.605493652 |
| *SRI* | -0.632558263 | 10.51131273 | -5.088554961 | 2.34E-06 | 5.47E-05 | 4.584241508 |
| *CFTR* | -0.723512419 | 8.657238197 | -5.078801773 | 2.43E-06 | 5.64E-05 | 4.547513599 |
| *SLC6A14* | 2.90276976 | 6.678076522 | 5.074821046 | 2.47E-06 | 5.70E-05 | 4.532533295 |
| *CCL3L3* | 0.646003571 | 7.460780072 | 5.071307529 | 2.51E-06 | 5.77E-05 | 4.51931605 |
| *EHHADH* | -0.61580583 | 7.795380972 | -5.069732976 | 2.52E-06 | 5.78E-05 | 4.513394332 |
| *MIF* | 0.679024153 | 9.008772099 | 5.068249021 | 2.54E-06 | 5.80E-05 | 4.507814177 |
| *MIR196A1* | -0.695303295 | 7.427135914 | -5.061918593 | 2.60E-06 | 5.87E-05 | 4.484018866 |
| *LANCL3* | -0.603993101 | 5.918319817 | -5.060005265 | 2.62E-06 | 5.90E-05 | 4.476829817 |
| *SATB2-AS1* | -0.779727706 | 6.574143592 | -5.055597718 | 2.67E-06 | 5.95E-05 | 4.460274272 |
| *REG3A* | 2.328148929 | 9.124764879 | 5.05478069 | 2.68E-06 | 5.97E-05 | 4.457206162 |
| *RAPGEFL1* | -0.796906008 | 7.916646259 | -5.047740159 | 2.75E-06 | 6.08E-05 | 4.430777771 |
| *AGMO* | -0.697912459 | 5.182293922 | -5.042807044 | 2.81E-06 | 6.17E-05 | 4.412271092 |
| *CYP2B6* | -1.193172279 | 7.034405831 | -5.037755497 | 2.86E-06 | 6.28E-05 | 4.393329529 |
| *HMGCS2* | -1.974267413 | 10.08099956 | -5.037018251 | 2.87E-06 | 6.29E-05 | 4.39056591 |
| *NPY6R* | -0.667660501 | 7.112185066 | -5.036413013 | 2.88E-06 | 6.30E-05 | 4.388297283 |
| *CLEC4E* | 1.166729216 | 4.3707536 | 5.030298034 | 2.95E-06 | 6.38E-05 | 4.365384067 |
| *AQP8* | -2.429076686 | 10.090834 | -5.027433222 | 2.98E-06 | 6.44E-05 | 4.354654258 |
| *IGFBP5* | 1.082790155 | 8.722781979 | 5.018449738 | 3.09E-06 | 6.59E-05 | 4.321027708 |
| *HCK* | 0.607966573 | 7.859640025 | 5.007706606 | 3.22E-06 | 6.82E-05 | 4.280854483 |
| *SOCS3* | 0.830989542 | 6.777332329 | 5.005661261 | 3.25E-06 | 6.85E-05 | 4.273210999 |
| *NCR3LG1* | -0.586282199 | 6.7428625 | -4.997986676 | 3.35E-06 | 7.03E-05 | 4.244545104 |
| *C1S* | 0.667762771 | 10.04181576 | 4.993209401 | 3.41E-06 | 7.12E-05 | 4.22671246 |
| *RPS6KA6* | -0.769123311 | 6.7230511 | -4.986304123 | 3.51E-06 | 7.27E-05 | 4.200951753 |
| *IFIT2* | 0.835292134 | 6.523925085 | 4.985644714 | 3.52E-06 | 7.27E-05 | 4.198492727 |
| *ADH1A* | -0.694185361 | 6.194340245 | -4.978818791 | 3.61E-06 | 7.44E-05 | 4.173047675 |
| *PSAT1* | 0.80465942 | 6.784706236 | 4.96886638 | 3.76E-06 | 7.69E-05 | 4.135979977 |
| *MIER3* | -0.758605612 | 9.227441825 | -4.965830469 | 3.80E-06 | 7.77E-05 | 4.124680318 |
| *SLC17A4* | -1.153813788 | 8.320619978 | -4.956622267 | 3.94E-06 | 7.96E-05 | 4.090429108 |
| *SLC6A6* | 0.609314426 | 7.405017878 | 4.947756122 | 4.08E-06 | 8.13E-05 | 4.057481179 |
| *NFKBIZ* | 0.671944777 | 8.084886165 | 4.943960956 | 4.14E-06 | 8.21E-05 | 4.043387075 |
| *SATB2* | -0.785113434 | 9.487416086 | -4.923915366 | 4.48E-06 | 8.76E-05 | 3.969036767 |
| *PADI2* | -1.057012369 | 8.88640301 | -4.923149303 | 4.50E-06 | 8.78E-05 | 3.966198504 |
| *COL4A2* | 0.623154349 | 7.566671404 | 4.920740515 | 4.54E-06 | 8.84E-05 | 3.95727544 |
| *ANXA1* | 0.999085469 | 8.875325811 | 4.916442631 | 4.62E-06 | 8.95E-05 | 3.941360099 |
| *CDHR1* | -0.623637332 | 6.47958829 | -4.915637924 | 4.63E-06 | 8.96E-05 | 3.938381025 |
| *PLEKHS1* | 0.906354984 | 6.494199599 | 4.912419631 | 4.69E-06 | 9.03E-05 | 3.926469239 |
| *MT-TL2* | -0.762417947 | 9.521823059 | -4.908470358 | 4.76E-06 | 9.14E-05 | 3.911857463 |
| *MMP3* | 2.487155151 | 7.023377637 | 4.906774583 | 4.79E-06 | 9.18E-05 | 3.905585206 |
| *GBP1* | 0.831709596 | 9.943506711 | 4.895488537 | 5.01E-06 | 9.44E-05 | 3.863869781 |
| *CASP5* | 0.985365899 | 9.033875826 | 4.891451165 | 5.09E-06 | 9.53E-05 | 3.848959091 |
| *RAVER2* | -0.649331529 | 8.853929216 | -4.878482836 | 5.36E-06 | 9.88E-05 | 3.801108626 |
| *SLAMF7* | 0.796420893 | 8.189029857 | 4.875598282 | 5.42E-06 | 9.95E-05 | 3.790474307 |
| *LINC01123* | -0.610893582 | 7.9551199 | -4.875598277 | 5.42E-06 | 9.95E-05 | 3.790474287 |
| *FCER1G* | 0.680790293 | 8.259881543 | 4.875054978 | 5.43E-06 | 9.96E-05 | 3.78847171 |
| *PRO2012* | -0.688639938 | 6.219470153 | -4.873703813 | 5.46E-06 | 1.00E-04 | 3.78349188 |
| *IFITM2* | 0.586870563 | 8.732236346 | 4.872092649 | 5.49E-06 | 0.000100397 | 3.777554753 |
| *PLIN2* | -0.64536173 | 7.637513839 | -4.869805609 | 5.54E-06 | 0.000100943 | 3.769128812 |
| *CXCL2* | 0.990619823 | 6.349248732 | 4.865685547 | 5.63E-06 | 0.000102258 | 3.753954903 |
| *FPR1* | 1.165586291 | 6.443157945 | 4.861473429 | 5.72E-06 | 0.000103386 | 3.738448974 |
| *SLC9A2* | -0.75325884 | 8.728954645 | -4.85612271 | 5.84E-06 | 0.000105156 | 3.718761813 |
| *NNMT* | 0.660191859 | 6.871773863 | 4.847987038 | 6.03E-06 | 0.000107379 | 3.688849873 |
| *SAA2* | 0.971307065 | 5.931825834 | 4.833722688 | 6.38E-06 | 0.000111796 | 3.636469392 |
| *ARL14* | -0.754551943 | 8.071320672 | -4.832737088 | 6.40E-06 | 0.000111972 | 3.632853185 |
| *PARP14* | 0.596524722 | 8.756582605 | 4.826779895 | 6.55E-06 | 0.000113486 | 3.611004376 |
| *ENTPD5* | -0.871150836 | 8.965771686 | -4.82374141 | 6.63E-06 | 0.000114492 | 3.599865872 |
| *CD163* | 0.58518305 | 7.943795506 | 4.82315872 | 6.64E-06 | 0.000114666 | 3.597730269 |
| *ST3GAL4* | 0.849214364 | 6.660665031 | 4.808193618 | 7.04E-06 | 0.000119224 | 3.542929469 |
| *PARP9* | 0.67129634 | 8.53020404 | 4.803982126 | 7.15E-06 | 0.000120572 | 3.527523871 |
| *SLC30A10* | -1.396108654 | 8.04484766 | -4.799232371 | 7.29E-06 | 0.0001221 | 3.510158031 |
| *RNA5SP403* | 1.034871759 | 9.578305917 | 4.794295858 | 7.43E-06 | 0.000123766 | 3.492119183 |
| *BST2* | 0.666089397 | 7.277811064 | 4.794192275 | 7.43E-06 | 0.000123766 | 3.491740783 |
| *STOM* | 0.619892153 | 9.023313608 | 4.792800389 | 7.47E-06 | 0.000124201 | 3.486656465 |
| *CYR61* | 0.997176216 | 6.602763291 | 4.785621417 | 7.68E-06 | 0.000127426 | 3.460445601 |
| *RNA5SP493* | 0.755544045 | 7.898615149 | 4.781339495 | 7.81E-06 | 0.000129247 | 3.444822157 |
| *IGKV2D-26* | 1.116002662 | 9.589760426 | 4.778361267 | 7.90E-06 | 0.000130589 | 3.433959965 |
| *TAGLN* | 0.671223496 | 7.890426689 | 4.77785015 | 7.92E-06 | 0.000130743 | 3.432096188 |
| *PLAU* | 1.070014719 | 7.707836562 | 4.776941077 | 7.94E-06 | 0.000130976 | 3.428781539 |
| *CLDN1* | 0.949231497 | 6.373428651 | 4.775049038 | 8.00E-06 | 0.00013171 | 3.421883912 |
| *IGLV7-46* | 1.138557257 | 7.857062227 | 4.773433041 | 8.05E-06 | 0.000132162 | 3.415993798 |
| *PHLPP2* | -0.92521148 | 7.347537006 | -4.764247865 | 8.34E-06 | 0.000135826 | 3.382535516 |
| *CXCL8* | 1.418185221 | 6.775908172 | 4.763206949 | 8.38E-06 | 0.000136142 | 3.378746047 |
| *CXCL9* | 1.465287105 | 7.238877596 | 4.759121814 | 8.51E-06 | 0.000137441 | 3.363878405 |
| *ELOVL5* | 0.623691332 | 8.822512886 | 4.75277881 | 8.72E-06 | 0.000139588 | 3.340807141 |
| *ACSF2* | -0.792709559 | 7.484519647 | -4.751260663 | 8.77E-06 | 0.000140213 | 3.335287709 |
| *NCF2* | 0.767572653 | 7.765185578 | 4.749143765 | 8.84E-06 | 0.000140848 | 3.327593043 |
| *COL1A2* | 0.797374156 | 9.879719476 | 4.747853984 | 8.89E-06 | 0.000141387 | 3.322905766 |
| *PLEK* | 1.034602696 | 8.278279406 | 4.746748929 | 8.93E-06 | 0.000141893 | 3.318890368 |
| *SLC51A* | -0.923902524 | 8.098356375 | -4.745519 | 8.97E-06 | 0.000142373 | 3.314421819 |
| *SLC5A8* | 0.756310117 | 4.778833669 | 4.721648 | 9.83E-06 | 0.000152839 | 3.227820028 |
| *TMED6* | -0.594064156 | 5.403404278 | -4.705130984 | 1.05E-05 | 0.000159321 | 3.168038551 |
| *HCAR3* | 1.503604268 | 5.642992621 | 4.704983985 | 1.05E-05 | 0.000159321 | 3.167507023 |
| *SAMD9L* | 0.867891575 | 7.94920804 | 4.692284022 | 1.10E-05 | 0.000165434 | 3.121620529 |
| *CA2* | -0.797996917 | 11.40807405 | -4.686413615 | 1.13E-05 | 0.000168254 | 3.100433289 |
| *COL4A1* | 0.812431209 | 7.512608419 | 4.679538466 | 1.16E-05 | 0.000170948 | 3.075638523 |
| *SRGN* | 0.691419519 | 9.974373405 | 4.676069723 | 1.17E-05 | 0.000172792 | 3.063136425 |
| *G0S2* | 0.682840727 | 7.214394988 | 4.672289722 | 1.19E-05 | 0.000174204 | 3.049518366 |
| *ATP6V0D2* | -0.655417245 | 5.752730683 | -4.670725627 | 1.19E-05 | 0.000174699 | 3.043885262 |
| *SNX10* | 0.884892923 | 7.08943276 | 4.668537784 | 1.20E-05 | 0.000175067 | 3.036007489 |
| *CXCL10* | 1.558720362 | 7.548515767 | 4.662669503 | 1.23E-05 | 0.000178036 | 3.01488773 |
| *EXPH5* | -0.783979038 | 6.836766998 | -4.65646908 | 1.26E-05 | 0.000181401 | 2.992588747 |
| *SLC7A5* | 0.607484061 | 7.309642313 | 4.65576815 | 1.27E-05 | 0.000181445 | 2.990068994 |
| *HSD17B2* | -1.033718365 | 9.378337348 | -4.652977812 | 1.28E-05 | 0.000182594 | 2.980040191 |
| *MTMR11* | -0.596631873 | 8.595655599 | -4.641500899 | 1.34E-05 | 0.000187995 | 2.938826305 |
| *CXCL6* | 0.802422113 | 6.416132801 | 4.640261748 | 1.34E-05 | 0.00018863 | 2.934379903 |
| *RNA5SP413* | 0.669700242 | 9.292772645 | 4.638469137 | 1.35E-05 | 0.00018972 | 2.927948723 |
| *BNIP3* | 0.620966441 | 6.734484698 | 4.63580569 | 1.37E-05 | 0.000191159 | 2.918395911 |
| *ETNK1* | -0.766989271 | 9.649648222 | -4.633493619 | 1.38E-05 | 0.000191784 | 2.910105864 |
| *PLAUR* | 0.664158121 | 8.393703491 | 4.629786367 | 1.40E-05 | 0.000193531 | 2.896818189 |
| *FFAR2* | 0.953764771 | 5.657117607 | 4.629185079 | 1.40E-05 | 0.000193859 | 2.894663597 |
| *CXCL5* | 1.166907511 | 7.700300786 | 4.623515882 | 1.43E-05 | 0.000196914 | 2.874356944 |
| *PPARG* | -0.720050689 | 7.866276199 | -4.623058561 | 1.43E-05 | 0.000197141 | 2.872719465 |
| *RNA5SP191* | 1.152051303 | 8.33257522 | 4.613083783 | 1.49E-05 | 0.000202597 | 2.837026698 |
| *SAA2-SAA4* | 0.693251814 | 4.878340691 | 4.611399324 | 1.50E-05 | 0.000203547 | 2.831003505 |
| *C4BPA* | 1.736353531 | 7.185173485 | 4.608417087 | 1.51E-05 | 0.000204434 | 2.820342849 |
| *IGKV3OR2-268* | 0.730887688 | 9.69165983 | 4.607123932 | 1.52E-05 | 0.000205076 | 2.815721399 |
| *HNF1A-AS1* | -0.996049235 | 8.129364368 | -4.605484552 | 1.53E-05 | 0.000205769 | 2.809863678 |
| *COL1A1* | 0.894418984 | 9.266742999 | 4.603326172 | 1.54E-05 | 0.000206885 | 2.802153309 |
| *SLC5A1* | 0.746084181 | 7.884495491 | 4.603244424 | 1.54E-05 | 0.000206885 | 2.801861321 |
| *FABP1* | -0.72504546 | 11.95608476 | -4.590061051 | 1.62E-05 | 0.00021494 | 2.754811531 |
| *SERPINA1* | 0.634260937 | 9.243251159 | 4.583075842 | 1.67E-05 | 0.000219085 | 2.729913384 |
| *TUBAL3* | -0.587928016 | 6.877111382 | -4.578712689 | 1.70E-05 | 0.000221772 | 2.714372305 |
| *IGKV3D-11* | 0.725589148 | 11.3468125 | 4.578395486 | 1.70E-05 | 0.000221772 | 2.713242791 |
| *SNORA64* | 0.7839884 | 8.186854207 | 4.575508062 | 1.72E-05 | 0.000223809 | 2.702963163 |
| *RN7SL394P* | 0.656946076 | 7.19659449 | 4.572694909 | 1.73E-05 | 0.000225194 | 2.692951519 |
| *ACSM3* | -0.606589172 | 7.645848553 | -4.556364934 | 1.84E-05 | 0.000234881 | 2.634905035 |
| *CCL11* | 0.804346083 | 7.577856058 | 4.55628955 | 1.85E-05 | 0.000234881 | 2.634637354 |
| *ALDH1A2* | 0.664578052 | 5.506685886 | 4.547403233 | 1.91E-05 | 0.000240264 | 2.603100554 |
| *CES2* | -0.615383844 | 9.203347108 | -4.539548489 | 1.97E-05 | 0.000245625 | 2.575254278 |
| *TNIP3* | 1.885112203 | 6.356646024 | 4.538561591 | 1.97E-05 | 0.000246144 | 2.571757539 |
| *RNA5SP242* | 0.741931308 | 8.865784064 | 4.525977489 | 2.07E-05 | 0.000255738 | 2.527208593 |
| *ADH1C* | -0.919313076 | 10.10537975 | -4.519933515 | 2.12E-05 | 0.000259979 | 2.505837822 |
| *KRTAP5-3* | 0.594858817 | 8.54390959 | 4.515501722 | 2.15E-05 | 0.000262959 | 2.490178068 |
| *CSF3R* | 0.82178654 | 6.055126184 | 4.509507886 | 2.20E-05 | 0.000266643 | 2.46901304 |
| *IFNG* | 0.723804189 | 4.824163681 | 4.506081972 | 2.23E-05 | 0.000269173 | 2.456923031 |
| *SELENBP1* | -0.845301613 | 10.94218828 | -4.495357596 | 2.32E-05 | 0.000277069 | 2.41911149 |
| *GUCA2A* | -1.299472575 | 9.813730853 | -4.491922611 | 2.35E-05 | 0.000279552 | 2.407011695 |
| *DAPP1* | 0.677317423 | 6.352748412 | 4.491884882 | 2.35E-05 | 0.000279552 | 2.406878823 |
| *RNF157* | -0.593961005 | 6.589910106 | -4.484393922 | 2.42E-05 | 0.000285034 | 2.380510682 |
| *TINAG* | -0.776677105 | 7.061032173 | -4.481737656 | 2.44E-05 | 0.000286866 | 2.37116683 |
| *SRD5A3* | 0.659729288 | 7.452040968 | 4.479063049 | 2.47E-05 | 0.000289023 | 2.361761742 |
| *MMP9* | 0.938939133 | 7.807773121 | 4.476011053 | 2.49E-05 | 0.000291462 | 2.351033611 |
| *SNORD29* | 0.8793078 | 8.720974576 | 4.465324927 | 2.60E-05 | 0.000300626 | 2.313504474 |
| *PP7080* | -0.80520944 | 10.34136749 | -4.456682197 | 2.68E-05 | 0.000307732 | 2.283190273 |
| *PCK1* | -1.476126868 | 8.235835104 | -4.449450487 | 2.76E-05 | 0.000313063 | 2.257851829 |
| *PFKFB3* | 0.727269034 | 7.554108751 | 4.441996691 | 2.83E-05 | 0.000319698 | 2.231760706 |
| *CTSK* | 0.8213207 | 7.945628981 | 4.436250167 | 2.89E-05 | 0.000325918 | 2.211663369 |
| *RGS5* | 0.873137496 | 8.357290285 | 4.434998186 | 2.91E-05 | 0.000326715 | 2.207286856 |
| *CWH43* | -1.163562215 | 7.168649259 | -4.434794843 | 2.91E-05 | 0.000326804 | 2.206576106 |
| *TIMP1* | 0.90749985 | 10.11328591 | 4.425172308 | 3.02E-05 | 0.00033564 | 2.172964312 |
| *B4GALNT2* | -1.137278468 | 8.595722938 | -4.413896496 | 3.14E-05 | 0.000347345 | 2.13363285 |
| *ICAM1* | 0.742516362 | 7.684199419 | 4.406858633 | 3.23E-05 | 0.000354687 | 2.109114223 |
| *IGKV3D-20* | 0.727460235 | 11.21054862 | 4.401798735 | 3.29E-05 | 0.000360047 | 2.091500907 |
| *SULF1* | 0.779933254 | 7.319262752 | 4.395970334 | 3.36E-05 | 0.00036616 | 2.071227467 |
| *RNA5SP85* | 0.674475705 | 8.512173402 | 4.363861639 | 3.78E-05 | 0.000399268 | 1.959830366 |
| *APOBEC3B* | -0.67271648 | 7.095160962 | -4.357227266 | 3.88E-05 | 0.000406049 | 1.936874609 |
| *SNORA80E* | 0.764302412 | 10.11123546 | 4.355388341 | 3.90E-05 | 0.000407808 | 1.930515439 |
| *C4B* | 0.73841591 | 8.052190167 | 4.355310337 | 3.91E-05 | 0.000407808 | 1.930245731 |
| *SLC26A2* | -1.543248916 | 10.3104928 | -4.347801003 | 4.02E-05 | 0.000416288 | 1.904294913 |
| *IL1B* | 1.483549471 | 7.157142066 | 4.33489352 | 4.21E-05 | 0.000432498 | 1.85975261 |
| *CD55* | 0.940481283 | 9.273391015 | 4.332496493 | 4.25E-05 | 0.000435196 | 1.851489595 |
| *FAM160A1* | -0.605975206 | 7.438807168 | -4.327072058 | 4.33E-05 | 0.000441223 | 1.832800787 |
| *SNORA73A* | 0.629294937 | 11.30541376 | 4.322451087 | 4.41E-05 | 0.000447992 | 1.816891404 |
| *PTGDR* | -0.6717123 | 7.710043428 | -4.313646061 | 4.55E-05 | 0.000460094 | 1.786605598 |
| *FPR2* | 1.218717619 | 5.181153138 | 4.306719796 | 4.67E-05 | 0.000468069 | 1.762808512 |
| *RN7SL38P* | 0.667088922 | 8.403015706 | 4.300119632 | 4.78E-05 | 0.000477061 | 1.740153622 |
| *PDPN* | 0.686671721 | 6.897026774 | 4.292990482 | 4.91E-05 | 0.000486962 | 1.71570694 |
| *PCSK1* | 0.79853618 | 5.702023946 | 4.286049887 | 5.04E-05 | 0.000495682 | 1.691930781 |
| *ERO1L* | 0.589310819 | 9.287996765 | 4.284435766 | 5.07E-05 | 0.000497773 | 1.686404732 |
| *NR1H4* | -0.627302109 | 5.932083646 | -4.274952661 | 5.24E-05 | 0.00051229 | 1.653964594 |
| *PLA2G2A* | 1.200814711 | 9.91249598 | 4.27397117 | 5.26E-05 | 0.00051343 | 1.650609608 |
| *NXPE4* | -1.266617902 | 9.360274626 | -4.273729692 | 5.27E-05 | 0.000513506 | 1.649784247 |
| *IRF4* | 0.610993499 | 7.917135648 | 4.268654725 | 5.37E-05 | 0.000520489 | 1.632444897 |
| *GPX8* | 0.641095548 | 6.085048766 | 4.268022244 | 5.38E-05 | 0.000521257 | 1.63028483 |
| *MYO1A* | -0.609423017 | 9.398848589 | -4.263451856 | 5.47E-05 | 0.000528695 | 1.61468177 |
| *PECAM1* | 0.715064184 | 8.987994277 | 4.255615917 | 5.63E-05 | 0.000541532 | 1.58795437 |
| *C4BPB* | 0.977007683 | 6.074742081 | 4.25222342 | 5.70E-05 | 0.000547131 | 1.576392441 |
| *SAA1* | 1.470910404 | 4.692068163 | 4.242556496 | 5.90E-05 | 0.000562078 | 1.543478098 |
| *S100A9* | 0.912953448 | 6.641787716 | 4.233019401 | 6.11E-05 | 0.000576886 | 1.511051445 |
| *PHLDA1* | 0.593004751 | 7.71641267 | 4.230830717 | 6.16E-05 | 0.00057968 | 1.503616209 |
| *S100A12* | 1.232971507 | 5.167913253 | 4.190744222 | 7.12E-05 | 0.000648697 | 1.367863056 |
| *MAOA* | -0.686709 | 10.09835896 | -4.185313895 | 7.26E-05 | 0.000657168 | 1.349535635 |
| *RNA5SP352* | 0.623571759 | 10.62662425 | 4.181288347 | 7.37E-05 | 0.000663174 | 1.335959019 |
| *VLDLR* | -0.62184153 | 6.100261658 | -4.178464081 | 7.44E-05 | 0.000668669 | 1.326438783 |
| *SPARC* | 0.61198313 | 9.583580167 | 4.175522202 | 7.52E-05 | 0.000674496 | 1.316526403 |
| *RN7SL63P* | 0.615440443 | 7.355591935 | 4.170344848 | 7.66E-05 | 0.000684033 | 1.299092515 |
| *HCAR2* | 1.066939438 | 6.174006488 | 4.158900849 | 7.99E-05 | 0.000707098 | 1.260605281 |
| *IL1RN* | 0.986564969 | 6.60915596 | 4.147364855 | 8.32E-05 | 0.000728002 | 1.221876531 |
| *MMP10* | 1.18367321 | 5.417203105 | 4.137435305 | 8.63E-05 | 0.000748666 | 1.188595715 |
| *VWF* | 0.76369363 | 6.710312429 | 4.136149068 | 8.66E-05 | 0.000751286 | 1.184288358 |
| *A1CF* | -0.63166255 | 8.073226072 | -4.131752727 | 8.80E-05 | 0.000761491 | 1.169572311 |
| *TRIB2* | 0.600258411 | 6.992036954 | 4.126772529 | 8.96E-05 | 0.00077124 | 1.152913956 |
| *TLR2* | 0.595395648 | 5.935477497 | 4.126580159 | 8.97E-05 | 0.00077124 | 1.152270751 |
| *SULT1A2* | -0.636582049 | 7.421184134 | -4.125658904 | 9.00E-05 | 0.000772715 | 1.149190725 |
| *STEAP4* | 0.973430491 | 5.701969199 | 4.120462205 | 9.17E-05 | 0.000781604 | 1.131824869 |
| *XPNPEP2* | -0.606911238 | 5.905246444 | -4.119110302 | 9.21E-05 | 0.000784531 | 1.127309495 |
| *RN7SL448P* | 0.677971023 | 4.99307102 | 4.115632877 | 9.33E-05 | 0.000791088 | 1.115699204 |
| *CD274* | 1.025984561 | 6.17032835 | 4.110380001 | 9.50E-05 | 0.000801512 | 1.098173014 |
| *MMP2* | 0.706787443 | 8.483489179 | 4.096707814 | 9.98E-05 | 0.000831327 | 1.052623174 |
| *KIAA1324L* | -0.641022882 | 6.143643886 | -4.094475174 | 0.000100566 | 0.000836474 | 1.045194238 |
| *MMP7* | 1.103476714 | 4.891606445 | 4.088257061 | 0.000102819 | 0.000851163 | 1.02451769 |
| *TMC5* | 0.602617261 | 8.982750756 | 4.087696257 | 0.000103024 | 0.000852054 | 1.022653894 |
| *CXCR1* | 1.026483395 | 5.16355085 | 4.085219631 | 0.000103937 | 0.000857193 | 1.014424946 |
| *THY1* | 0.71283744 | 7.419785955 | 4.081799126 | 0.00010521 | 0.000865006 | 1.003065113 |
| *OSM* | 0.649166902 | 5.703809113 | 4.077547389 | 0.000106813 | 0.000876233 | 0.988953234 |
| *SLC4A4* | -0.787306119 | 9.244152291 | -4.077318347 | 0.0001069 | 0.000876639 | 0.988193294 |
| *COL6A3* | 0.823700346 | 8.400257791 | 4.060315769 | 0.000113554 | 0.000916647 | 0.931857382 |
| *TCN1* | 0.907868344 | 4.499256782 | 4.057852957 | 0.00011455 | 0.000919571 | 0.923709806 |
| *KYNU* | 0.855243506 | 5.927055102 | 4.041143354 | 0.000121534 | 0.000960483 | 0.86851518 |
| *CA1* | -1.680329571 | 10.22172499 | -4.037952233 | 0.000122913 | 0.00096843 | 0.857991218 |
| *SLC26A3* | -1.04730708 | 12.27917906 | -4.037466704 | 0.000123124 | 0.000969438 | 0.856390471 |
| *SERPINA3* | 1.026510766 | 6.135466363 | 4.03301991 | 0.000125075 | 0.000981481 | 0.841735623 |
| *FMO5* | -0.670922384 | 7.990539244 | -4.029097993 | 0.00012682 | 0.000992949 | 0.828819296 |
| *IFIT3* | 0.704099212 | 6.48299899 | 4.02596369 | 0.000128231 | 0.001001034 | 0.818502772 |
| *BIRC3* | 0.659503801 | 9.492329068 | 4.009033266 | 0.000136119 | 0.001043208 | 0.762867251 |
| *SERPINE1* | 0.664840745 | 6.056058297 | 4.008664636 | 0.000136296 | 0.00104422 | 0.761657594 |
| *GPR128* | 0.766815507 | 7.222846646 | 4.006425359 | 0.000137375 | 0.001051103 | 0.754310987 |
| *UBD* | 0.972231551 | 7.720076677 | 4.002453793 | 0.000139309 | 0.001062758 | 0.741287721 |
| *SLC3A1* | -0.71811378 | 6.927410785 | -4.001462588 | 0.000139795 | 0.0010654 | 0.738038759 |
| *TNFAIP6* | 1.014833916 | 5.207378766 | 3.995607314 | 0.000142703 | 0.00108228 | 0.718857169 |
| *DHRS11* | -0.709032489 | 9.23231474 | -3.991480581 | 0.000144788 | 0.001094175 | 0.70534929 |
| *CCL2* | 0.780426715 | 8.056479937 | 3.985891504 | 0.000147658 | 0.001111693 | 0.687069424 |
| *CCL24* | 0.727588518 | 7.133734252 | 3.973357678 | 0.000154293 | 0.001149966 | 0.646137187 |
| *BGN* | 0.662121163 | 7.337001661 | 3.971643241 | 0.000155222 | 0.001154336 | 0.640544895 |
| *OTOP2* | -0.672495781 | 7.233324556 | -3.946294339 | 0.000169608 | 0.001237209 | 0.558046361 |
| *CXCR2* | 0.941146886 | 5.096366443 | 3.943564912 | 0.000171231 | 0.001245929 | 0.549184292 |
| *ACTG2* | 0.700512819 | 7.849670226 | 3.932602419 | 0.000177901 | 0.001281657 | 0.513631706 |
| *TMEM171* | -0.615780466 | 9.386135231 | -3.918789351 | 0.00018666 | 0.001327932 | 0.468928367 |
| *WNT5A* | 0.67885775 | 6.783826362 | 3.916204584 | 0.000188345 | 0.001337465 | 0.460574939 |
| *GREM1* | 1.041661571 | 7.716120218 | 3.910141073 | 0.000192353 | 0.001359922 | 0.440993405 |
| *CLEC6A* | 0.712812478 | 4.464791598 | 3.901989921 | 0.00019787 | 0.001388608 | 0.414702062 |
| *SLC44A5* | -0.6627538 | 5.210797295 | -3.895433475 | 0.000202418 | 0.001409909 | 0.393581117 |
| *COL5A2* | 0.642837511 | 7.091943458 | 3.889734099 | 0.000206452 | 0.001428191 | 0.375240529 |
| *GSTA1* | -0.706909533 | 5.338751248 | -3.88919294 | 0.000206839 | 0.001430444 | 0.373500017 |
| *UGT2A3* | -1.213289575 | 7.395660806 | -3.887359805 | 0.000208155 | 0.001436137 | 0.36760538 |
| *TMEM56* | -0.609639906 | 8.715104363 | -3.88633419 | 0.000208895 | 0.001440815 | 0.364308223 |
| *SPINK4* | 1.045448039 | 8.925095912 | 3.874728743 | 0.000217445 | 0.001484842 | 0.327039769 |
| *LRRC19* | -0.587140793 | 8.719774081 | -3.837063063 | 0.000247558 | 0.001640484 | 0.206604016 |
| *MT2A* | 0.592778314 | 10.97179666 | 3.833443991 | 0.000250652 | 0.001655812 | 0.195074113 |
| *ENKUR* | 0.686962622 | 4.296362569 | 3.828521119 | 0.000254921 | 0.001679727 | 0.17940236 |
| *SLC10A2* | -0.642272358 | 5.761652963 | -3.820465526 | 0.000262057 | 0.001714144 | 0.153787329 |
| *RNA5SP399* | 0.621315534 | 9.842402807 | 3.816823991 | 0.000265345 | 0.001730308 | 0.142220121 |
| *IGKV2D-40* | 1.421257572 | 5.993606172 | 3.809056158 | 0.00027249 | 0.001765542 | 0.11757106 |
| *GUCY1A3* | 0.675776338 | 6.739397013 | 3.782695377 | 0.000298134 | 0.001888125 | 0.034179043 |
| *IGLV6-57* | 0.672955949 | 7.157821655 | 3.776361829 | 0.000304629 | 0.001916763 | 0.014202232 |
| *SERPING1* | 0.617577623 | 8.19261231 | 3.774390925 | 0.000306678 | 0.001927053 | 0.007990454 |
| *ITGA5* | 0.638426726 | 6.968279964 | 3.76681245 | 0.000314679 | 0.001966723 | -0.015874118 |
| *FADS2* | 0.737276409 | 7.200464244 | 3.753022383 | 0.000329751 | 0.002039052 | -0.059213978 |
| *NPY1R* | -0.648762496 | 4.822191172 | -3.747525193 | 0.000335949 | 0.002066959 | -0.076460068 |
| *ACSL4* | 0.688166058 | 7.63753694 | 3.744715538 | 0.00033916 | 0.002082315 | -0.085267912 |
| *KCNJ15* | 0.616633582 | 5.034038962 | 3.7440429 | 0.000339933 | 0.002085961 | -0.087375853 |
| *FCGR2A* | 0.694381506 | 6.521346723 | 3.738557842 | 0.000346299 | 0.002121117 | -0.104555343 |
| *XDH* | 0.693668927 | 8.230103541 | 3.718601014 | 0.000370434 | 0.002243049 | -0.166913474 |
| *ACSL1* | 0.645255934 | 7.563962673 | 3.718451674 | 0.00037062 | 0.002243552 | -0.167379231 |
| *IRAK3* | 0.618210923 | 6.283572572 | 3.715355571 | 0.000374507 | 0.00226316 | -0.177032385 |
| *MIR197* | 0.587860603 | 6.853967341 | 3.710121498 | 0.000381166 | 0.002294127 | -0.193338631 |
| *TNC* | 1.011477896 | 6.745234644 | 3.701649469 | 0.000392184 | 0.00234245 | -0.219698401 |
| *EDNRA* | 0.606722944 | 5.924503939 | 3.684379344 | 0.00041559 | 0.002444659 | -0.273301845 |
| *TREM1* | 0.784025493 | 5.426277361 | 3.673184612 | 0.000431466 | 0.002515822 | -0.307954387 |
| *FBN1* | 0.60496671 | 6.937335925 | 3.672694021 | 0.000432174 | 0.002518065 | -0.309471283 |
| *GPR110* | 0.799709145 | 5.612522825 | 3.621552552 | 0.000512449 | 0.002873587 | -0.466814487 |
| *RNA5SP295* | -0.612821199 | 4.471610493 | -3.617307317 | 0.000519714 | 0.00290258 | -0.479805219 |
| *MMP1* | 1.627367252 | 7.466274415 | 3.6002814 | 0.000549846 | 0.003036662 | -0.53179681 |
| *RNU5D-1* | 1.438859595 | 7.616521808 | 3.598042872 | 0.000553929 | 0.00305559 | -0.53861954 |
| *ANPEP* | -0.917203077 | 11.2172303 | -3.591310967 | 0.000566381 | 0.003106632 | -0.559119232 |
| *TMIGD1* | -1.040446708 | 8.800412113 | -3.582974433 | 0.00058217 | 0.003167719 | -0.584467236 |
| *VTRNA1-1* | 0.589238751 | 3.775838458 | 3.571747274 | 0.000604093 | 0.003260508 | -0.618537814 |
| *NXPE1* | -0.706245195 | 9.645858895 | -3.568035484 | 0.000611512 | 0.003289912 | -0.629784965 |
| *IFI6* | 0.683355241 | 8.428430911 | 3.567687268 | 0.000612212 | 0.00329292 | -0.630839669 |
| *SLC2A3* | 0.717658713 | 8.364527061 | 3.556582516 | 0.000634951 | 0.003387084 | -0.664435839 |
| *PLA2G7* | 0.60587432 | 8.626416127 | 3.539909426 | 0.0006706 | 0.003531614 | -0.71473682 |
| *TDO2* | 0.851052635 | 5.907217722 | 3.535322445 | 0.000680735 | 0.003576754 | -0.728545371 |
| *MMP12* | 1.060856983 | 8.617143685 | 3.515127919 | 0.000727106 | 0.003759228 | -0.789184562 |
| *DEFB4B* | 0.98353287 | 5.399120365 | 3.499353076 | 0.000765391 | 0.003912922 | -0.836377316 |
| *HLA-DQB1* | 0.651198042 | 7.841545253 | 3.479155729 | 0.000817196 | 0.004119745 | -0.896575167 |
| *HEPACAM2* | -0.670789994 | 8.188656958 | -3.475559426 | 0.000826762 | 0.00415897 | -0.907267241 |
| *IGHV1OR15-1* | 1.10106638 | 8.006789935 | 3.452464796 | 0.000890755 | 0.00441044 | -0.975736507 |
| *HAVCR1* | -0.647110572 | 5.170697279 | -3.445722683 | 0.000910302 | 0.004484278 | -0.995661941 |
| *SLC9A3* | -0.70336762 | 9.516038857 | -3.445662441 | 0.000910478 | 0.004484278 | -0.995839849 |
| *TRIM22* | 0.602415964 | 8.118091471 | 3.420601821 | 0.000986755 | 0.004792105 | -1.069651131 |
| *TRPM6* | -0.704951813 | 6.36871398 | -3.40690879 | 0.00103094 | 0.004962222 | -1.109813461 |
| *DEFA5* | 1.213633057 | 7.532312426 | 3.398685993 | 0.001058362 | 0.005070132 | -1.133874084 |
| *LUM* | 0.599167456 | 8.594631418 | 3.393510422 | 0.001075974 | 0.00513971 | -1.148996176 |
| *MMRN1* | 0.594389966 | 5.515168926 | 3.378537989 | 0.00112849 | 0.005353263 | -1.192646667 |
| *VNN2* | 0.908357724 | 6.238102745 | 3.374188297 | 0.001144193 | 0.005411234 | -1.205300837 |
| *RN7SL655P* | 0.622647264 | 6.257726845 | 3.343649242 | 0.001260377 | 0.005854965 | -1.293803155 |
| *PTGS2* | 0.783818471 | 6.216802995 | 3.326380765 | 0.001330897 | 0.006105882 | -1.343580933 |
| *MNDA* | 0.616457136 | 5.620599632 | 3.310842176 | 0.001397506 | 0.006356416 | -1.388206829 |
| *FAP* | 0.872407126 | 4.464748775 | 3.30499532 | 0.001423371 | 0.006458935 | -1.404957959 |
| *DEFA6* | 1.266271003 | 7.296674708 | 3.251366872 | 0.001682496 | 0.007348622 | -1.557558075 |
| *GBA3* | -0.689780386 | 7.560520597 | -3.248699027 | 0.001696476 | 0.007397207 | -1.565100016 |
| *SELE* | 0.751883514 | 5.246580568 | 3.230065886 | 0.001797196 | 0.007722244 | -1.617644067 |
| *MT-TS1* | 0.906024197 | 10.66871672 | 3.205210727 | 0.00194028 | 0.008186128 | -1.687374443 |
| *COL12A1* | 0.803750887 | 6.551213779 | 3.169309868 | 0.002165851 | 0.008912842 | -1.787363474 |
| *SNORD61* | 0.925429351 | 7.544498983 | 3.165775894 | 0.002189333 | 0.008991118 | -1.797159231 |
| *LAMP3* | 0.607551539 | 7.405791005 | 3.144580242 | 0.002335231 | 0.009440571 | -1.855733951 |
| *SLC7A11* | 0.707565289 | 6.124207344 | 3.120481624 | 0.002512109 | 0.010013205 | -1.921960808 |
| *TRIM40* | 0.829008881 | 7.929447133 | 3.108745192 | 0.002602701 | 0.010289832 | -1.954071085 |
| *OSMR* | 0.615942555 | 6.983109796 | 3.072820071 | 0.002899267 | 0.01121313 | -2.051773608 |
| *SELP* | 0.635925997 | 6.277463352 | 3.051282294 | 0.003091829 | 0.011785764 | -2.109921675 |
| *CHP2* | -0.721775911 | 8.221842071 | -3.050874624 | 0.003095586 | 0.011794297 | -2.111019214 |
| *CD177* | -1.040613116 | 8.189334684 | -2.98043464 | 0.00381225 | 0.013917342 | -2.298919895 |
| *MME* | 0.819621916 | 5.567850651 | 2.970122218 | 0.003929221 | 0.014252829 | -2.32613653 |
| *CCL20* | 0.600377192 | 8.34740648 | 2.954626758 | 0.004111237 | 0.014745534 | -2.366891012 |
| *LIMD1-AS1* | -0.816428455 | 5.227279872 | -2.895132012 | 0.004884901 | 0.016969369 | -2.521781569 |
| *IL1A* | 0.650872382 | 4.504382191 | 2.878091174 | 0.005130039 | 0.017660164 | -2.565679421 |
| *CLDN8* | -0.594658391 | 3.96827475 | -2.872607602 | 0.0052113 | 0.017879369 | -2.579760839 |
| *IL6* | 0.670110594 | 4.772025927 | 2.840106587 | 0.005717744 | 0.01926525 | -2.662774797 |
| *IGKV6-21* | 0.660575668 | 8.576331784 | 2.798421751 | 0.006433448 | 0.021126947 | -2.768121653 |
| *CYP3A4* | -0.624628958 | 4.292068384 | -2.757899176 | 0.007207033 | 0.023199878 | -2.869310081 |
| *BMP3* | -0.594199584 | 7.490844415 | -2.67532973 | 0.009052399 | 0.027705908 | -3.071724408 |
| *PI15* | 0.775596393 | 5.234971982 | 2.652212504 | 0.009641087 | 0.02915847 | -3.127479872 |
| *REG4* | 0.619332397 | 7.7542953 | 2.505812115 | 0.014247352 | 0.039642882 | -3.471123169 |
| *ALDOB* | 0.812532075 | 8.057273828 | 2.492471835 | 0.014752667 | 0.040737637 | -3.501615112 |
| *FOS* | 0.587438031 | 8.875249555 | 2.479423036 | 0.01526245 | 0.041847843 | -3.531306601 |
| *SERPINB5* | 0.768909166 | 5.677789617 | 2.475011092 | 0.015438355 | 0.042171391 | -3.541315538 |
| *CKB* | -0.634486369 | 9.169941492 | -2.463192811 | 0.015918542 | 0.043219658 | -3.568051449 |
