# Supplementary material for: Identification of endoplasmic reticulum stress-associated genes and subtypes for predicting risk signature and depicting immune features in inflammatory bowel disease
Source: Heliyon. 2024 Sep 1;10(17):e37053. doi: 10.1016/j.heliyon.2024.e37053 (PMC11409092; doi:10.1016/j.heliyon.2024.e37053)
Supplement: Multimedia component 2 [file mmc2.docx]

Supplementary Table 2. The names of endoplasmic reticulum stress-related differentially expressed genes

| Gene names |
| --- |
| *TAP1* |
| *TAP2* |
| *ZC3H12A* |
| *TGM2* |
| *UGT1A1* |
| *DERL3* |
| *HLA-DRB3* |
| *HLA-DRB1* |
| *APOE* |
| *NOS2* |
| *SOD2* |
| *C1R* |
| *DYSF* |
| *PPARGC1A* |
| *XBP1* |
| *SCD* |
| *CFTR* |
| *C1S* |
| *CXCL8* |
| *COL1A2* |
| *PPARG* |
| *COL1A1* |
| *FABP1* |
| *SERPINA1* |
| *IFNG* |
| *MMP9* |
| *CTSK* |
| *ICAM1* |
| *SLC26A2* |
| *IL1B* |
| *FPR2* |
| *VWF* |
| *TLR2* |
| *MMP2* |
| *SERPINA3* |
| *SERPINE1* |
| *CCL2* |
| *XDH* |
| *EDNRA* |
| *PTGS2* |
| *IL6* |
| *FOS* |
