# Supplementary material for: Identification of endoplasmic reticulum stress-associated genes and subtypes for predicting risk signature and depicting immune features in inflammatory bowel disease
Source: Heliyon. 2024 Sep 1;10(17):e37053. doi: 10.1016/j.heliyon.2024.e37053 (PMC11409092; doi:10.1016/j.heliyon.2024.e37053)
Supplement: Multimedia component 3 [file mmc3.docx]

Supplementary Table 3. The names of 17 predictor genes

| Gene names |
| --- |
| *TAP1* |
| *ZC3H12A* |
| *UGT1A1* |
| *HLA-DRB3* |
| *APOE* |
| *NOS2* |
| *DYSF* |
| *SCD* |
| *CFTR* |
| *SERPINA1* |
| *IFNG* |
| *MMP9* |
| *ICAM1* |
| *VWF* |
| *CCL2* |
| *PTGS2* |
| *FOS* |
